# Dietary management of childhood diarrhea in low- and middle-income countries: a systematic review

**DOI:** 10.1186/1471-2458-13-S3-S17

**Published:** 2013-09-17

**Authors:** Michelle F Gaffey, Kerri Wazny, Diego G Bassani, Zulfiqar A Bhutta

**Affiliations:** 1 Centre for Global Child Health, The Hospital for Sick Children, Toronto, ON, Canada; 2 Dalla Lana School of Public Health, University of Toronto, Toronto, ON, Canada; 3 Department of Paediatrics, University of Toronto, Toronto, ON, Canada; 4 Division of Women and Child Health, Aga Khan University Hospital, Karachi, Pakistan

## Abstract

**Background:**

Current WHO guidelines on the management and treatment of diarrhea in children strongly recommend continued feeding alongside the administration of oral rehydration solution and zinc therapy, but there remains some debate regarding the optimal diet or dietary ingredients for feeding children with diarrhea.

**Methods:**

We conducted a systematic search for all published randomized controlled trials evaluating food-based interventions among children under five years old with diarrhea in low- and middle-income countries. We classified 29 eligible studies into one or more comparisons: reduced versus regular lactose liquid feeds, lactose-free versus lactose-containing liquid feeds, lactose-free liquid feeds versus lactose-containing mixed diets, and commercial/specialized ingredients versus home-available ingredients. We used all available outcome data to conduct random-effects meta-analyses to estimate the average effect of each intervention on diarrhea duration, stool output, weight gain and treatment failure risk for studies on acute and persistent diarrhea separately.

**Results:**

Evidence of low-to-moderate quality suggests that among children with acute diarrhea, diluting or fermenting lactose-containing liquid feeds does not affect any outcome when compared with an ordinary lactose-containing liquid feeds. In contrast, moderate quality evidence suggests that lactose-free liquid feeds reduce duration and the risk of treatment failure compared to lactose-containing liquid feeds in acute diarrhea. Only limited evidence of low quality was available to assess either of these two approaches in persistent diarrhea, or to assess lactose-free liquid feeds compared to lactose-containing mixed diets in either acute or persistent diarrhea. For commercially prepared or specialized ingredients compared to home-available ingredients, we found low-to-moderate quality evidence of no effect on any outcome in either acute or persistent diarrhea, though when we restricted these analyses to studies where both intervention and control diets were lactose-free, weight gain in children with acute diarrhea was shown to be greater among those fed with a home-available diet.

**Conclusions:**

Among children in low- and middle-income countries, where the dual burden of diarrhea and malnutrition is greatest and where access to proprietary formulas and specialized ingredients is limited, the use of locally available age-appropriate foods should be promoted for the majority of acute diarrhea cases. Lactose intolerance is an important complication in some cases, but even among those children for whom lactose avoidance may be necessary, nutritionally complete diets comprised of locally available ingredients can be used at least as effectively as commercial preparations or specialized ingredients. These same conclusions may also apply to the dietary management of children with persistent diarrhea, but the evidence remains limited.

## Background

While childhood diarrhea mortality has declined steadily since the 1980s, diarrheal disease remains the third leading cause of death among children under-five globally. An estimated 800,000 under-five deaths were attributable to diarrhea in 2010, accounting for 11% of all under-five deaths, with about 80% of these diarrheal deaths occurring in the African and Southeast Asian WHO regions [1].

Current WHO guidelines on the management and treatment of diarrhea in children strongly recommend continued feeding alongside administration of oral rehydration solutions, plus zinc therapy [2,3]. The benefit of early feeding of children with diarrhea has been known since the late 1940s [4], with clinical and community-based studies since then providing further evidence to support early and continued feeding during diarrhea [5-7]. A recent systematic review found no evidence to suggest that early compared to delayed feeding in acute diarrhea increases the risk of complications [7], and continued feeding from the early stage of a diarrheal episode can mitigate the consequences of reduced absorption and increased loss of nutrients, and thereby also limit the cumulative and longer-term effects of diarrhea morbidity on child growth [8].

Continued feeding is now widely accepted as a key component of appropriate treatment for childhood diarrhea, but with the exception of consensus on continued breastfeeding, there remains some debate regarding the optimal diet or dietary ingredients for hastening recovery and maintaining nutritional status in children with diarrhea [8,9]. Lactose malabsorption is a common complication of diarrhea [10], especially among malnourished children [11], but limiting milk intake among young children can promote further nutritional deficiency if substitute sources of protein and energy are not consumed sufficiently. Commercial preparations of soy-based or other lactose-free formulations may be effective, but they are not routinely available to households in the settings in which most diarrhea morbidity and mortality occur, where the use of locally available foods to comprise appropriate treatment diets is far more feasible. Earlier reviews of the literature have narratively and/or quantitatively summarized evidence for the effectiveness of several dietary regimens for managing childhood diarrhea [6,8,9,12-14]. In the present review we sought to update some of these analyses with particular reference to children in low- and middle-income countries, and then to explicitly compare the use of costly commercial or specialized preparations with diets of locally available foods on which the home management of childhood diarrhea in low- and middle-income countries could more feasibly be based.

## Objective

This review aimed to assess the relative effectiveness of several approaches to the dietary management of childhood diarrhea in hastening recovery and improving nutritional status in children with diarrhea in low- and middle-income countries.

## Methods

We used the PICO (Population, Intervention, Comparison, Outcome) approach to frame our research question, establish our inclusion criteria and develop our search strategy. The population of interest was children under five years of age with diarrhea in low- and middle-income countries. We sought to find and include all randomized controlled trials in this population that evaluated continued feeding with one specified diet compared to at least one other specified diet. For analysis, individual studies were then grouped into broader sets of dietary comparisons, as described below. We considered three continuous outcomes of interest, ideally measured from the start of the dietary intervention (following initial correction of dehydration) until the resolution of diarrhea, but measured over shorter time periods in some studies: duration of diarrhea, stool output, and weight change. We also considered the proportion of participants experiencing treatment failure during the study period. The definition of treatment failure was that used by the individual study authors and typically included the need for a change in clinical management, including a change of diet. Specific criteria for treatment failure included sustained diarrhea beyond a certain period, worsening of diarrhea, or recurrent dehydration. If the study authors did not explicitly define treatment failure but data were available for an outcome consistent with the need for a change in clinical management, we used these.

### Search strategy

We searched the following electronic databases initially in December 2011 and finally in September 2012, with no restriction on date or language: Medline, Embase, AMED, LILACS, WHOLIS, African Index Medicus, Index Medicus for the Eastern Mediterranean Region, Index Medicus for South-East Asia Region, and Western Pacific Region Index Medicus. Our search inputs combined various terms for the four concepts of *diarrhea*, *child/infant/newborn*, *feeding/food/diet* and *trial/comparative study*. The specific inputs used for Medline, Embase and AMED are given in Additional File 1. We also examined reference lists of previous reviews related to dietary management of childhood diarrhea to identify any potentially relevant publications not found through the electronic search.

### Search results and study selection

All titles and abstracts returned by the electronic search as well as the reference lists of previous related reviews were independently screened for relevance in duplicate. The full-text reports of all titles screened as relevant were then independently examined in duplicate, to determine each study’s eligibility with respect to the review inclusion criteria – i.e. randomized controlled trials that evaluated food-based dietary interventions among children under five years of age with diarrhea in low- and middle-income countries. We based country eligibility on the World Bank’s country classification by income [15]. Trials evaluating probiotics, micronutrient supplementation, oral rehydration solution formulations, or other non-food-based nutritional treatment of diarrhea were not included unless the effects of the food-based components included in these trials could be isolated. Studies that met the inclusion criteria but did not report on outcomes of interest to this review were included as eligible but did not contribute to the quantitative analyses.

### Data abstraction

Data from each eligible study were independently abstracted in duplicate using a data collection form to capture information on study characteristics, participant characteristics, components of the diets compared, any co-interventions, quantitative results reported for each outcome of interest (overall and by sub-group, if presented), key conclusions and comments on study limitations made by study authors, and funding sources. The two sets of abstracted data were compared and any discrepancies were resolved through discussion and by consulting a third reviewer where necessary. Attempts were made to contact study authors by email for missing information on outcomes. Where studies reported weight change or stool output for separate periods during the study but not for the overall study period, we used data from the earliest post-rehydration period reported [16]. In two studies that reported standard deviations for the mean baseline and endline weights but not for the mean change in weight [17,18], we imputed standard deviations for the mean change assuming a correlation of 0.75 between the baseline and endline standard deviations [19]. Alternative imputed estimates assuming higher and lower correlation did not affect the pooled estimates to which these data contributed. The abstracted data for all studies included in the quantitative data synthesis are presented in Additional File 2**.**

### Intervention comparisons

We identified the comparisons for this review based on the literature on lactose reduction and avoidance in diarrhea and on home-based dietary management of diarrhea, but we were also constrained by the results of our search strategy. Once the detail of each diet evaluated in each eligible study was abstracted, we classified each study into one or more possible dietary comparisons. The following four comparisons of interest were finally identified both for their substantive focus and for the number of eligible studies with which they were compatible:

1) Liquid feeds: reduced lactose versus regular lactose

2) Liquid feeds: lactose-free versus lactose-containing

3) Lactose-free liquid feeds versus lactose-containing mixed diets

4) Diets that include commercial preparations or specialized ingredients versus diets comprised of home-available ingredients

The comparison definitions, the classification of included studies by comparison, and details of the diets evaluated in each study are given in Table 1.

**Table 1:** Comparison definitions, study diets, and inventory of available outcome data

Study ID	Intervention diet	Control diet	Available outcome data			
	
			Duration	Stool output	Weight change	Treatment failure
**Comparison 1. Liquid feeds: Reduced lactose versus Regular lactose** ***Comparison of diets that include lactose-containing liquid feeds*, *where the intervention liquid feed contains less lactose than the control liquid feed***						

Bhatnagar 1998	Fermented formula + rice-lentil-oil gruel	Milk formula + rice-lentil-oil gruel				Y
Chew 1993	Gradually increased concentration of milk formula	Full strength milk formula	Y	Y	Y	Y
Ibanez 1986	Acidified milk formula	Milk formula		Y		
Lifshitz 1991	Diluted cow's milk	Cow's milk formula				Y
Pichaipat 1986	Gradually increased concentration of milk formula	Full strength milk formula				Y
Ransome 1984	Gradually increased concentration of cow's milk	Full-strength cow's milk	Y			Y
Singh 1987	Yogurt	Milk	Y			
Touhami 1989	Half strength milk or milk formula	Full strength milk or milk formula	Y	Y	Y	Y
Touhami 1992	Fermented milk formula + cereals + vegetable soup	Milk formula + cereals + vegetable soup		Y	Y	Y

**Comparison 2. Liquid feeds: Lactose-free versus Lactose-containing** ***Comparison of diets that include liquid feeds*, *where the intervention liquid feed is lactose-free and the control liquid feed is lactose-containing***						

Brown 1991	i) Lactose-hydrolyzed powdered milk + corn syrup solids; ii) Lactose-hydrolyzed powdered milk + corn syrup solids, with wheat noodles	i) Powdered milk + corn syrup solids; ii) Powdered milk + corn syrup solids, with wheat noodles	Y	Y	Y	Y
Fayad 1999	Soy-based formula with sucrose	Soy-based formula with lactose	Y		Y	Y
Haffejee 1990	Soy-based formula	Cow's milk formula	Y			
Lifshitz 1991	i) Lactose-free sodium caseinate formula; ii) Lactose-free casein hydrolysate formula; iii) Soy-based formula	i) Diluted cow's milk; ii) Cow's milk formula				Y
Lozano 1994	Lactose-free casein-based formula plus non-milk food	Cow's milk formula plus same non-milk food	Y			Y
Naidoo 1981	Soy-based formula	Cow's milk formula				Y
Penny 1989	Lactose-hydrolyzed powdered milk + corn syrup solids	Powdered milk + corn syrup solids				Y
Rajah 1988	i) Lactose-free casein-based formula; ii) Soy-based formula; iii) Lactose-free hydrolyzed whey formula	Cow's milk formula		Y		Y
Romer 1989	Lactose-free semi-elemental formula	Cow's milk			Y	Y
Simakachorn 2004	Soy-based formula + rice gruel	Cow's milk formula + rice gruel	Y	Y	Y	Y

**Comparison 3. Lactose-free liquid feeds versus Lactose-containing mixed diets** ***Comparison of lactose-free liquid feeds with mixed diets containing lactose***						

Alarcon 1991	Soy-based formula	Potato flour + milk powder + carrot flour + veg oil + sugar				Y
Bhutta 1991	Soy-based formula	Rice + lentils + cottonseed oil + yogurt		Y	Y	Y
Bhutta 1994	Soy-based formula	Rice + lentils + cottonseed oil + yogurt + diluted buffalo milk	Y	Y	Y	Y
Brown 1991	Lactose-hydrolyzed powdered milk + corn syrup solids	Powdered milk + corn syrup solids, with wheat noodles	Y	Y	Y	Y

**Comparison 4. Commercially prepared or specialized ingredients versus Home-available ingredients** ***Comparison of commercial preparations or specialized ingredients with diets comprised of home-available ingredients***						

Alarcon 1991	Soy-based formula	i) Wheat flour + pea flour + carrot flour + veg oil + cane sugar; ii) Potato flour + milk powder + carrot flour + veg oil + sugar				Y
Alarcon 1992	Rice + vegetable oil + soy protein isolate + corn syrup solids	Rice + vegetable oil +white beans				Y
Bhan 1988	Cow's milk formula	Rice + lentil + sugar + coconut oil	Y		Y	Y
Bhutta 1991	Soy-based formula	Rice + lentils + cottonseed oil + yogurt		Y	Y	Y
Bhutta 1994	Soy-based formula	Rice + lentils + cottonseed oil + yogurt + diluted buffalo milk	Y	Y	Y	Y
Brown 1991	i) Lactose-hydrolyzed powdered milk + corn syrup solids; ii) Lactose-hydrolyzed powdered milk + corn syrup solids, with wheat noodles	i) Powdered milk + corn syrup solids; ii) Powdered milk + corn syrup solids, with wheat noodles	Y	Y	Y	Y
Carias 1999	Soy-based formula	Lactose-free chicken-based formula		Y	Y	
Godard 1989	Hydrolyzed lactalbumin + dextrin-maltrose + sunflower oil + carrots	Chicken + dextrin-maltrose + sunflower oil + carrots	Y			
Grange 1994	Soy-based formula	Fermented maize flour + toasted cowpea flour + palm oil + sugar				Y
Lifshitz 1991	i) Lactose-free sodium caseinate formula; ii) Lactose-free casein hydrolysate formula; iii) Soy-based formula; iv) Cow's milk formula	Diluted cow's milk				Y
Maulen-Radovan 1994	Soy-based formula	Rice + chicken + carrots + beans + vegetable oil		Y	Y	Y
Nurko 1997	i) Elemental formula: ii) Soy-based formula	Chicken + sugar + minerals + cooking oil	Y	Y	Y	Y
Penny 1989	Lactose-hydrolyzed powdered milk + corn syrup solids	Powdered milk + corn syrup solids				Y
Romer 1989	Lactose-free semi-elemental formula	Cow's milk			Y	Y
Santosham 1990	i) Soy-based formula; ii) Rice-based formula	Liquefied boiled rice	Y	Y	Y	Y

### Quality assessment

We applied the CHERG adaptation of the GRADE technique [20] to assess the overall quality of evidence for each outcome for each comparison. First, each study was graded on a four-point continuum from “high” to “very low”, with the initial “high” score for randomized, controlled design being adjusted downward if necessary, as indicated by the assessment of the study’s methods (including randomization and allocation concealment procedures, blinding, completeness of outcome ascertainment and outcome reporting, and other criteria) on risk of bias. Secondly, an overall evidence score on the same scale was then assigned for each outcome for each comparison, taking into account the quality of the studies included, the volume and consistency of results across studies, the size of the pooled effect estimate, and the strength of evidence for that estimate as indicated by its p-value.

### Quantitative analysis

For each meta-analysis, we first stratified studies by participants’ duration of diarrhea at study enrolment: acute (duration of 7 days or less) or persistent (duration of 14 days or more). No studies focused specifically on prolonged diarrhea (duration between 7 and 14 days). Assuming the variety of diets evaluated in each comparison were unlikely to produce the same, fixed intervention effect, we decided *a priori* to use random-effects models for all meta-analyses to instead estimate the average intervention effect. For the three continuous outcomes (duration of diarrhea, stool output, weight change) we estimated the standardized mean difference, which allows for data on the same outcome but measured on different scales to be pooled in one meta-analysis. For the dichotomous outcome of treatment failure we estimated the risk ratio. We assessed the presence of heterogeneity by examining the extent of confidence interval overlap in the forest plots and we quantified heterogeneity using the I-squared statistic, with an I-squared value of 50% or greater indicating moderate heterogeneity [21]. All meta-analyses were conducted in Review Manager 5.1.

## Results

### Identification of studies

**Figure 1:**
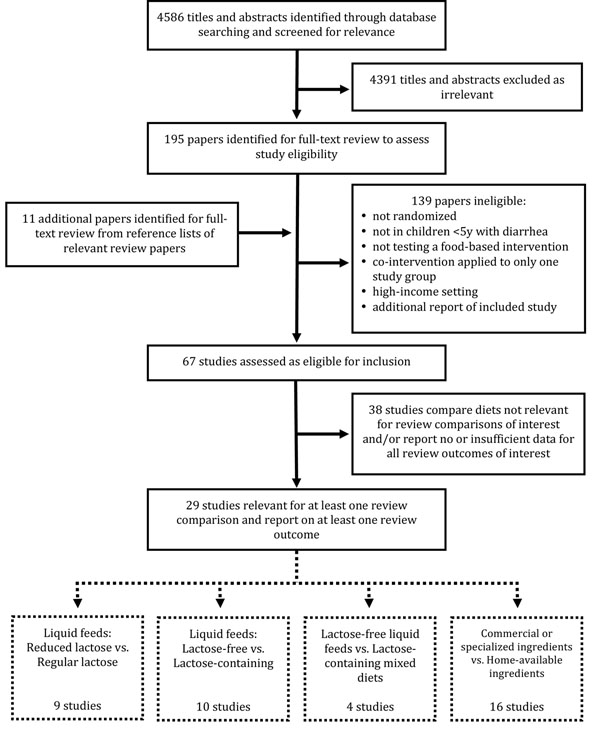
Flow diagram showing identification of studies

Our electronic search strategy returned 4586 titles and abstracts from which 195 papers were screened as relevant and retrieved for full-text examination (Figure 1). An additional 11 relevant titles were identified from the reference lists of previous reviews related to our topic. After excluding 139 papers that either did not meet our inclusion criteria, did not apply co-interventions equally across all study groups or were additional reports of already included studies, the remaining 67 studies were assessed as eligible for inclusion. Of these, 38 studies either included dietary comparisons that were not consistent with any of the four comparisons on which this review focused or did not report sufficient data for any four outcomes of interest, or both (Additional File 3). The remaining 29 studies were included in the quantitative data synthesis (Table 1).

### Quantitative data synthesis

The results from the meta-analyses conducted for each comparison and outcome are presented below, stratified by duration of diarrhea at study enrolment. The forest plots generated for all meta-analyses are presented in Additional File 4.

### 1. Liquid feeds: reduced lactose versus regular lactose

#### Acute diarrhea

Eight trials among children with acute diarrhea compared diets comprised partially or wholly of lactose-containing liquid feeds, where the lactose content of one liquid feed was reduced compared to the lactose content of the other. Five studies [22-26] compared diluted to undiluted milk products, and three studies [27-29] compared acidified or fermented milk products to regular milk products. One study allowed non-milk complementary foods in addition to the intervention and control liquid feeds, with both comparison groups receiving the identical complementary foods [27]. All studies excluded severely malnourished children.

The pooled results from four studies reporting on diarrhea duration [22,25,26,29], three studies reporting on stool output [22,26,28], two studies reporting on weight change [22,26] and six studies reporting on treatment failure [22-27] showed no statistically significant effects of reduced lactose liquid feeds on any outcome (Table 2). The overall quality of evidence was assessed as low for the outcome of duration and moderate for the stool output, weight change and treatment failure outcomes.

**Table 2:** Quality assessment of studies on reduced lactose versus regular lactose liquid feeds

		QUALITY ASSESSMENT					SUMMARY OF FINDINGS		
**Number of studies**	**Diarrhea mode**	**Design**	**Limitations**	**Consistency**	**Generalizability to population of interest**	**Overall quality of evidence**	**Number of events in intervention group**	**Number of events in control group**	**Effect size (95% CI)**

**OUTCOME: DURATION OF DIARRHEA**									**Standardized Mean Difference**

4	Acute	RCT	Study quality ranges from low to high	Heterogeneous (I^2^= 82%)	Infants and young children (≤36m) with acute diarrhea, not severely malnourished, in LMICs	Low	-	-	-0.49 [-1.04, 0.07]
0	Persistent	RCT	No studies	-	-	-	-	-	-

**OUTCOME: STOOL OUTPUT**									**Standardized Mean Difference**

3	Acute	RCT	Study quality ranges from moderate to high	Consistent (I^2^= 41%)	Infants with acute diarrhea, not severely malnourished, in LMICs	Moderate	-	-	-0.18 [-0.56, 0.19]
1	Persistent	RCT	Single study of moderate quality	-	Non-malnourished, non-breastfed infants with persistent diarrhea in LMICs	Low	-	-	-0.25 (-0.73, 0.24)

**OUTCOME: WEIGHT GAIN**									**Standardized Mean Difference**

2	Acute	RCT	Only two studies, of moderate to high quality	Consistent (I^2^= 0%)	Infants with acute diarrhea, not severely malnourished, in LMICs	Moderate	-	-	-0.02 [-0.29, 0.25]
1	Persistent	RCT	Single study of moderate quality	-	Non-malnourished, non-breastfed infants with persistent diarrhea in LMICs	Low	-	-	0.39 (-0.09, 0.87)

**OUTCOME: TREATMENT FAILURE**									**Risk Ratio**

6	Acute	RCT	Study quality ranges from moderate to high; two studies report zero event counts in both groups	Consistent (I^2^= 0%)	Infants and young children (≤48m) with acute diarrhea, not severely malnourished, in LMICs	Moderate	30	31	1.08 [0.71, 1.64]
1	Persistent	RCT	Single study of moderate quality	-	Non-malnourished, non-breastfed infants with persistent diarrhea in LMICs	Low	4	15	0.27 [0.10, 0.74]

#### Persistent diarrhea

A single study among well-nourished children with persistent diarrhea compared a fermented milk formula with a regular milk formula, with both study groups also receiving cereals and vegetable soup [30]. Data on diarrhea duration were not reported, and no statistically significant effect was shown for either stool output or weight change. A large and statistically significant reduction in the risk of treatment failure was reported for the use of yogurt compared to milk (Risk ratio (RR): 0.27; 95%CI: 0.10 to 0.74; p=0.01), but the overall quality of evidence was assessed as low given that no other studies were included in the analysis.

### 2. Liquid feeds: lactose-free versus lactose-containing

#### Acute diarrhea

Eight trials among children with acute diarrhea compared diets comprised partially or wholly of liquid feeds, where the liquid feed in one study group was lactose-free and the liquid feed in the other contained lactose. Six studies compared cow’s milk or cow’s milk-based formula with soy-based, casein-based or whey-based formulas [16,23,31-34], one study compared regular milk to milk in which at least 95% of the lactose had been hydrolyzed [35], and one study compared soy-based formulas with and without added lactose [36]. Three studies allowed non-milk complementary foods in addition to the intervention and control liquid feeds, with complementary foods given identically across comparison groups [16,32,36]. All studies excluded severely malnourished children.

**Figure 2:**
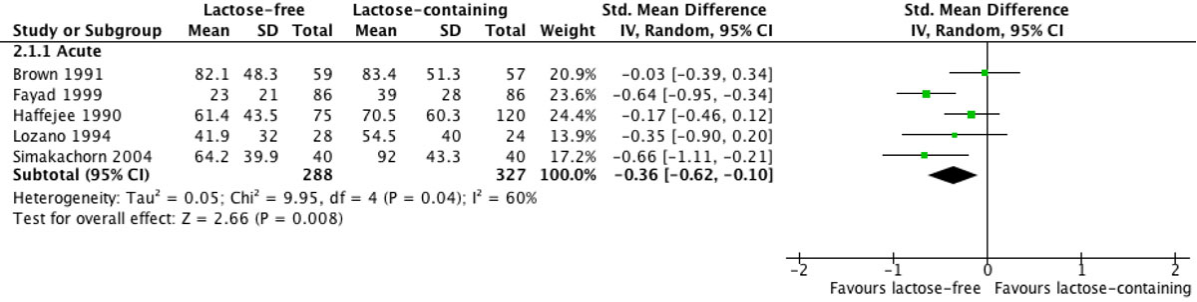
Effect of lactose-free versus lactose-containing liquid feeds on duration of diarrhea

**Figure 3:**
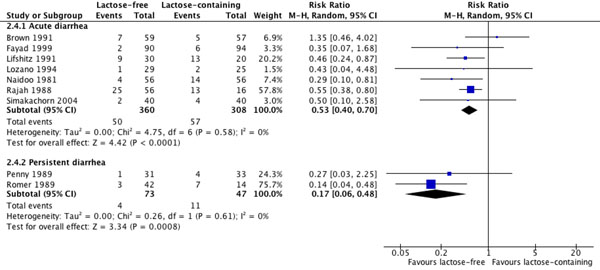
Effect of lactose-free versus lactose-containing liquid feeds on risk of treatment failure

The pooled result from five studies [16,31,32,35,36] showed a statistically significant effect of lactose-free liquid feeds on reducing diarrhea duration (SMD: -0.36; 95%CI: -0.62 to -0.10; p=0.008) (Table 3). Effect sizes and their statistical significance varied across studies but were consistent in direction (Figure 2). No effect of lactose-free liquid feeds was shown in the pooled results from three studies on stool output [16,34,35] or from three studies on weight change [16,35,36], but the evidence from seven studies [16,23,32-36] showed a statistically significant reduction of 47% in the risk of treatment failure (RR: 0.53; 95%CI: 0.40 to 0.70; p<0.0001). Effect sizes and their statistical significance varied across studies but were consistent in direction with the exception of one study (Figure 3). The overall quality of evidence for all four outcomes was assessed as moderate.

**Table 3:** Quality assessment of studies on lactose-free versus lactose-containing liquid feeds

		QUALITY ASSESSMENT					SUMMARY OF FINDINGS		
**Number of studies**	**Diarrhea mode**	**Design**	**Limitations**	**Consistency**	**Generalizability to population of interest**	**Overall quality of evidence**	**Number of events in intervention group**	**Number of events in control group**	**Effect size (95% CI)**

**OUTCOME: DURATION OF DIARRHEA**									**Standardized Mean Difference**

5	Acute	RCT	Study quality ranges from moderate to high	Heterogeneous (I^2^= 60%)	Infants and young children (≤24m) with acute diarrhea, without severe malnutrition, in LMICs	Moderate	-	-	-0.36 [-0.62, -0.10]
0	Persistent	RCT	No studies	-	-	-	-	-	-

**OUTCOME: STOOL OUTPUT**									**Standardized Mean Difference**

3	Acute	RCT	Study quality ranges from moderate to high	Heterogeneous (I^2^= 76%)	Infants and young children (≤24m) with acute diarrhea, without severe malnutrition, in LMICs	Moderate	-	-	-0.26 [-0.80, 0.28]
0	Persistent	RCT	No studies	-	-	-	-	-	-

**OUTCOME: WEIGHT GAIN**									**Standardized Mean Difference**

3	Acute	RCT	Study quality ranges from moderate to high	Heterogeneous (I^2^= 41%)	Infants and young children (≤24m) with acute diarrhea, not severely malnourished, in LMICs	Moderate	-	-	0.05 [-0.22, 0.33]
1	Persistent	RCT	Single study of low quality	-	Infants and young children (≤22m) with persistent diarrhea, not dehydrated or severely malnourished, in LMICs	Very low	-	-	0.90 (0.07, 1.73)

**OUTCOME: TREATMENT FAILURE**									**Risk Ratio**

7	Acute	RCT	Study quality ranges from low to high	Consistent (I^2^= 0%)	Infants and young children (≤24m) with acute diarrhea, not severely malnourished, in LMICs	Moderate	50	57	0.53 [0.40, 0.70]
2	Persistent	RCT	Only two studies, of low and high quality	Consistent (I^2^= 0%)	Infants and young children (≤36m) with persistent diarrhea, not severely malnourished, in LMICs	Low	4	11	0.17 [0.06, 0.48]

#### Persistent diarrhea

Two studies compared lactose-free to lactose-containing liquid feeds among children with persistent diarrhea and without severe malnutrition: one compared lactase-treated milk with regular milk [37] and the other compared lactose-free semi-elemental formulas to regular milk [38]. No data were available for the outcomes of diarrhea duration or stool output. Data on weight gain were available only from the study of semi-elemental formulas and showed a large and statistically significant effect of these formulas compared to milk (SMD: 0.90; 95%CI: 0.07 to 1.73; p=0.03), but this evidence was assessed as very low quality because of the study’s high and differential attrition rates across study groups (Additional File 2). The pooled result from both studies showed a large and statistically significant effect of lactose-free liquid feeds in reducing the risk of treatment failure (RR: 0.17; 95%CI: 0.06 to 0.48; p=0.0008), but the overall quality of this evidence was assessed as low.

### 3. Lactose-free liquid feeds versus lactose-containing mixed diets

#### Acute diarrhea

Two trials compared lactose-free liquid feeds to lactose-containing mixed diets among non-malnourished children with acute diarrhea: one compared lactose-hydrolyzed milk to a combination of regular milk and wheat noodles [35] and the other compared soy-based formula to a mixture of potato and carrot flours, milk powder, sugar and vegetable oil [39]. Data available from the single study reporting on duration, stool output and weight change reported a statistically significant increased duration (SMD: 0.75; 95%CI: 0.22 to 1.28; p=0.006) and increased stool output (SMD: 0.62; 95%CI: 0.09 to 1.14; p=0.02) with lactose-free liquid feeds compared to mixed diets (Table 4), but the overall quality of evidence for all three outcomes was assessed as low given that no other studies were included in the analysis. The pooled result from both studies showed no significant difference in the risk of treatment failure, with the quality of evidence for this outcome also assessed as low given the low number of studies included and the heterogeneity of their effect estimates.

**Table 4:** Quality assessment of studies on lactose-free liquid feeds versus lactose-containing mixed diets

		QUALITY ASSESSMENT					SUMMARY OF FINDINGS		
**Number of studies**	**Diarrhea mode**	**Design**	**Limitations**	**Consistency**	**Generalizability to population of interest**	**Overall quality of evidence**	**Number of events in intervention group**	**Number of events in control group**	**Effect size (95% CI)**

**OUTCOME: DURATION OF DIARRHEA**									**Standardized Mean Difference**

1	Acute	RCT	Single study of high quality	-	Non-malnourished infants and young children (≤24m) with acute diarrhea in LMICs	Low	-	-	0.75 [0.22, 1.28]
1	Persistent	RCT	Single study of moderate quality	-	Malnourished weaned infants and young children (≤36m) with persistent diarrhea in LMICs	Low	-	-	0.28 [-0.35, 0.90]

**OUTCOME: STOOL OUTPUT**									**Standardized Mean Difference**

1	Acute	RCT	Single study of high quality	-	Non-malnourished infants and young children (≤24m) with acute diarrhea in LMICs	Low	-	-	0.62 [0.09, 1.14]
2	Persistent	RCT	Only two studies, both of moderate quality	Heterogeneous (I^2^= 50%)	Malnourished weaned infants and young children (≤36m) with persistent diarrhea in LMICs	Low	-	-	0.22 [-0.33, 0.78]

**OUTCOME: WEIGHT GAIN**									**Standardized Mean Difference**

1	Acute	RCT	Single study of high quality	-	Non-malnourished infants and young children (≤24m) with acute diarrhea in LMICs	Low	-	-	0.21 [-0.31, 0.72]
2	Persistent	RCT	Only two studies, both of moderate quality	Heterogeneous (I^2^= 94%)	Malnourished weaned infants and young children (≤36m) with persistent diarrhea in LMICs	Low	-	-	-0.35 [-2.00, 1.31]

**OUTCOME: TREATMENT FAILURE**									**Risk Ratio**

2	Acute	RCT	Only two studies, ranging from moderate to high quality	Heterogeneous (I^2^= 60%)	Non-malnourished infants and young children (≤24m) with acute diarrhea in LMICs	Low	7	3	1.79 [0.15, 20.66]
2	Persistent	RCT	Only two studies, both of moderate quality	Heterogeneous (I^2^= 72%)	Malnourished weaned infants and young children (≤36m) with persistent diarrhea in LMICs	Low	6	7	1.25 [0.07, 23.66]

#### Persistent diarrhea

Two trials conducted among malnourished, weaned children with persistent diarrhea compared soy-based formula with a mixture of rice, lentils, and yogurt, with the later trial also adding diluted buffalo milk to the mixed diet [40,41]. No statistically significant effects of the soy-based formula were shown in the single study reporting on duration [41] or in the pooled results from both studies for stool output, weight gain or treatment failure. The quality of evidence was assessed as low for all outcomes, given the low number and the heterogeneity of the study-specific estimates included.

### 4. Commercially prepared or specialized ingredients versus home-available ingredients

#### Acute diarrhea

Nine trials conducted among children with acute diarrhea compared diets that contained commercial preparations or specialized ingredients with diets comprised of home-available foods and ingredients.

The commercial/specialized diets under study included proprietary cow’s milk-based, rice-based, soy-based, or lactose-free casein-based formulas in seven studies [17,23,39,42-45]; lactose-hydrolyzed cow’s milk with or without wheat noodles in one study [35]; and locally available foods combined with soy protein isolate in one study [46]. The home-available diets under study included regular cow’s milk with [35] or without wheat noodles [23,35] in two studies; chicken-containing formulations in two studies [17,44]; rice and lentils or beans in two studies [42,46]; porridges made from wheat, potato, maize or carrot flours in two studies [39,43]; and boiled rice alone in one study [45]. All studies excluded children with severe malnutrition.

The pooled results from three studies reporting on duration [35,42,45], four studies reporting on stool output [17,35,44,45], five studies reporting on weight gain [17,35,42,44,45] and eight studies reporting on treatment failure [23,35,39,42-46] showed no statistically significant effect of commercial/specialized diets compared to home available diets on any outcome (Table 5). The overall quality of evidence was assessed as moderate for all four outcomes.

**Table 5:** Quality assessment of studies on commercial/specialized ingredients versus home-available ingredients

		QUALITY ASSESSMENT					SUMMARY OF FINDINGS		
**Number of studies**	**Diarrhea mode**	**Design**	**Limitations**	**Consistency**	**Generalizability to population of interest**	**Overall quality of evidence**	**Number of events in intervention group**	**Number of events in control group**	**Effect size (95% CI)**

**OUTCOME: DURATION OF DIARRHEA**									**Standardized Mean Difference**

3	Acute	RCT	Study quality ranges from moderate to high	Consistent (I^2^= 0%)	Infants and young children (≤24m) with acute diarrhea, without severe malnutrition, in LMICs	Moderate	-	-	-0.13 [-0.36, 0.09]
3	Persistent	RCT	Studies of moderate quality	Consistent (I^2^= 0%)	Malnourished infants and young children (≤36m) with persistent diarrhea in LMICs	Moderate	-	-	-0.03 [-0.37, 0.32]

**OUTCOME: STOOL OUTPUT**									**Standardized Mean Difference**

4	Acute	RCT	Study quality ranges from moderate to high	Consistent (I^2^= 0%)	Infants and young children (≤36m) with acute diarrhea, without severe malnutrition, in LMICs	Moderate	-	-	0.15 [-0.06, 0.36]
3	Persistent	RCT	Studies of moderate quality	Consistent (I^2^= 25%)	Malnourished infants and young children (≤36m) with persistent diarrhea in LMICs	Moderate	-	-	0.16 [-0.23, 0.54]

**OUTCOME: WEIGHT GAIN**									**Standardized Mean Difference**

5	Acute	RCT	Study quality ranges from moderate to high	Heterogeneous (I^2^= 58%)	Infants and young children (≤36m) with acute diarrhea, without severe malnutrition, in LMICs	Moderate	-	-	-0.09 [-0.40, 0.23]
4	Persistent	RCT	Study quality ranges from low to moderate	Heterogeneous (I^2^= 88%)	Malnourished infants and young children (≤36m) with persistent diarrhea in LMICs	Low	-	-	0.04 (-0.90, 0.97)

**OUTCOME: TREATMENT FAILURE**									**Risk Ratio**

8	Acute	RCT	Study quality ranges from moderate to high	Heterogeneous (I^2^= 49%)	Infants and young children (≤36m) with acute diarrhea, without severe malnutrition, in LMICs	Moderate	34	25	0.82 [0.37, 1.79]
5	Persistent	RCT	Study quality ranges from low to high; nutritional status ranges from non-malnourished to severely malnourished	Heterogeneous (I^2^= 67%)	Infants and young children (≤36m) with persistent diarrhea in LMICs	Low	21	22	0.55 [0.17, 1.74]

When we restricted these analyses to only those studies in which both the intervention and control diets were both lactose-free, no statistically significant effects of the commercial/specialized diets were shown with respect to duration, stool output or treatment failure, but the pooled result from three studies reporting on weight gain [17,44,45] showed a statistically significant reduction in weight gain with commercial/specialized diets compared to home-available diets (SMD: -0.30; 95%CI: -0.55 to -0.04; p=0.02) (Additional File 4).

#### Persistent diarrhea

Six studies conducted among children with persistent diarrhea compared diets containing commercial preparations or specialized ingredients with diets comprised of home-available foods and ingredients.

The commercial/specialized diets included proprietary rice-based, soy-based, lactose-free whey-based, or amino acid-based formulas in four studies [18,38,40,41]; lactose-hydrolyzed milk in one study [37]; and locally available foods combined with lactalbumin hydrolysate in one study [47]. The home-available diets included a rice, lentil and yogurt mixture with or without diluted buffalo milk in two studies [40,41]; chicken-containing formulations in two studies [18,47]; and cow’s milk in two studies [37,38].

The pooled result from three studies reporting on duration [18,41,47] and on stool output [18,40,41] among moderately or severely malnourished children showed no statistically significant effects of commercial/specialized diets compared to home-available diets. The overall quality of evidence for both outcomes was assessed as moderate. No statistically significant effects were shown from the pooled results of four studies reporting on weight gain [18,37,40,41] or the five studies reporting on treatment failure [18,37,38,40,41]. The pooled results for weight gain and treatment failure included two studies conducted among children who were normal or moderately malnourished children [37,38], two studies among moderately or severely malnourished children [40,41], and one study among only severely malnourished children [18]. Stratifying the studies by nutritional status, there continued to be no statistically significant effects among moderately or severely malnourished children, or among only severely malnourished children, but a significant increase in weight gain and a significant decrease in treatment failure risk was shown among normal or moderately malnourished children [37,38]. However, the overall quality of evidence for this stratum was assessed as very low, as in the analysis of the same two studies in the comparison of lactose-free with lactose-containing liquid feeds in persistent diarrhea, reported above.

When we restricted these analyses to only those two studies in which the commercial/specialized diet and the home-available diet were both lactose-free [18,47], no statistically significant effects were shown with respect to any of the four outcomes, though the overall quality of evidence for these outcomes was low. Both studies were conducted among severely malnourished children.

## Discussion

We used data from 29 randomized controlled trials conducted in low- and middle-income countries to assess the evidence for several approaches to the dietary management of childhood diarrhea, including lactose reduction and avoidance and the use of home-available foods. We found evidence of low-to-moderate quality suggesting that among children with acute diarrhea, diluting or fermenting a lactose-containing liquid feed does not affect diarrhea duration, stool output, weight gain or risk of treatment failure compared with an ordinary lactose-containing liquid feed given at full strength. In contrast, we found moderate quality evidence suggesting that lactose-free liquid feeds reduce duration and the risk of treatment failure compared to lactose-containing liquid feeds in acute diarrhea. Only limited evidence of low quality was available to assess either of these two approaches in persistent diarrhea, or to assess lactose-free liquid feeds compared to lactose-containing mixed diets in either acute or persistent diarrhea. Our analyses of all studies on the use of commercially prepared or specialized ingredients compared to home-available ingredients found low-to-moderate quality evidence of no effect on any outcome in either acute or persistent diarrhea. When we restricted these analyses to studies where both the commercial/specialized and the home-available diets were lactose-free, weight gain in children with acute diarrhea was shown to be greater among those fed with a home-available diet.

The present review supplements the existing literature on dietary management of childhood diarrhea in at least two ways. First, we have focused our analyses exclusively on children in low- and middle-income countries, where the global burden of diarrhea incidence and mortality is concentrated and where financial and logistic barriers to accessing proprietary infant formulas and specialized ingredients are greatest, including in hospital settings. While severely malnourished children were excluded from the majority of trials we analyzed, nearly all included moderately malnourished children in their study populations and only one trial was restricted to well-nourished children. The results of this review thus generalize directly to the settings in which most children affected by diarrhea live. Secondly, we have attempted to disaggregate the effects of various approaches to dietary management by duration of diarrhea at study enrolment, recognizing that persistent post-infectious diarrhea may constitute a related but distinct pathophysiology from that of acute diarrhea, involving prolonged intestinal mucosal injury and delayed mucosal regeneration which increase the risk of chronic malnutrition and growth failure and may also inhibit neurodevelopment [48]. Previously reviewed evidence from community-based cohort studies in Asia, Africa and Latin America suggests that persistent diarrhea accounts for 3% to 20% of all childhood diarrheal episodes [9,49] and between one third to one half of diarrhea mortality [9]. We therefore sought to separately investigate the evidence for the dietary management of this important subset of the total diarrheal burden.

A previous meta-analysis of studies on the use of non-human milks for treating acute diarrhea in children was published in 1994 [12]. In addition to our inclusion of new studies conducted since then, our analyses also differ from those in the previous review with respect to our narrower study population and by our inclusion of studies evaluating acidified or fermented milk products. Nonetheless, our finding of no apparent effect of reducing the lactose content of lactose-containing liquid feeds in acute diarrhea is consistent with the results of the previous review which showed no difference in duration, stool output or risk of treatment failure when comparing undiluted to diluted milks, though weight gain was shown to be higher among children fed undiluted milks. Similarly, our findings of increased duration and treatment failure risk among children with acute diarrhea consuming lactose-containing instead of lactose-free liquid feeds are consistent with the earlier analyses, though the previous review also found stool output to be higher among those fed lactose-containing diets. While transient lactase deficiency may be common among children with acute diarrhea, lactose intolerance does not always develop. The previous review concluded that feeding with lactose-containing milks can be safely and effectively continued in the majority of children with acute diarrhea, and the results of our analyses do not contradict this conclusion. However, the results of both reviews indicate that lactose intolerance is indeed an important complication in some cases. Stratified analyses in the previous review showed that the effects of avoiding lactose were largely restricted to those children with severe dehydration at enrolment or to those studies conducted earlier than 1985, before the current protocol of oral rehydration solution plus continued feeding was widely adopted. In the present review, none of the children included in the analyses on the lactose content of liquid feeds were severely dehydrated at enrolment and only two of the studies included in these analyses were conducted before 1985 [25,33], but most children were at least moderately malnourished. Thus, the particular characteristics associated with an increased risk of lactose intolerance among children with diarrhea remain unclear.

The potential benefits of feeding yogurt during diarrhea have been considered in previous reviews of the literature [8,12,14], but we were able to find and include only four randomized controlled trials of acidified or fermented milk products that were compatible with the comparisons and outcomes of interest to the present review, and these trials contributed to our analyses on the lactose content of liquid feeds. The two trials reporting on the outcome of duration in acute diarrhea [26,29] both showed significant decreases in duration with the yogurt feeds compared to the regular milk feeds (Additional File 4). We excluded from our study one recent trial from Brazil that compared yogurt to several lactose-free liquid feeds among children with persistent diarrhea and found a significant beneficial effect of the yogurt-based diet with respect to diarrhea duration and stool output [50]. However, the yogurt-based diet inadvertently included a far higher concentration of zinc than any of the lactose-free diets. While the therapeutic dose of zinc may have been largely responsible for these results, any additional effects of the yogurt-based feed remain unknown.

Additionally, beyond our analyses comparing the lactose-content of liquid feeds, we found insufficient evidence to meaningfully evaluate other strategies for reducing lactose intake rather than eliminating lactose from the diet altogether. The partial replacement of milk with staple foods such as cereals and legumes reduces the overall lactose content of the diet, can better maintain or increase energy and protein intake, and may also improve stool consistency due to higher fiber intake [35,51]. Results from a large multi-country cohort study conducted in Asia and Latin America in 1996 suggest that mixed diets comprised of local cereals, vegetable oil and milk or yogurt can be used effectively in 65% (95%CI: 61%-70%) of young, malnourished children with persistent diarrhea [52]. In the present review, we sought to evaluate the evidence for using lactose-containing mixed diets compared to lactose-free liquid feeds for managing childhood diarrhea, but we found too few randomized controlled trials to enable such an evaluation. Further experimental evidence on both the use of acidified or fermented milk products and the use of milk-staple mixtures may confirm these feasible approaches as effective for managing diarrhea in children for whom lactose intake is problematic but for whom the complete avoidance of milk would pose significant nutritional risk.

In our final set of analyses, we deliberately focused our inquiry on the pragmatic aspect of treating childhood diarrhea in low- and middle-income countries and we found no evidence to support the use of proprietary formulas or specialized ingredients over the use of locally produced and readily available foods in the treatment of either acute or persistent diarrhea. Moreover, this finding held even when we narrowed our analyses to studies in which both the commercial/specialized diets and the home-available diets were lactose-free. Notably, we found evidence suggesting that weight gain during acute diarrhea was better among children being fed lactose-free home-available ingredients than among those consuming lactose-free commercial/specialized diets. The practical implication of these findings is extremely important for resource-constrained settings generally and should inform efforts to improve home-based management of childhood diarrhea in particular.

In summary, the results of our analyses suggest that lactose reduction among children consuming lactose-containing liquid feeds has little effect on clinical outcomes in childhood diarrhea, but the avoidance of lactose altogether may be important in some cases. However, further evidence is needed to fully assess the potential clinical and nutritional effects of reducing lactose intake through the use of yogurt or milk-staple mixtures rather than by eliminating milk from the diet entirely. Finally, and perhaps most importantly, our analyses indicate that diets comprised of home-available ingredients can be used at least as effectively as diets comprised of commercial preparations or specialized ingredients for managing both acute and persistent diarrhea, even among those children from whom lactose avoidance may be necessary.

Our review suffers from some limitations. The dietary comparisons made in many existing studies on dietary management of childhood diarrhea were not compatible with the comparisons we ultimately identified for this review and were therefore not included (Additional File 3). Other approaches that have been evaluated by randomized controlled trial in low- and middle-income settings include the use of amylase-rich flours in cereal-based porridges to decrease viscosity and thus increase nutrient density and children’s nutrient intake [53-55], and incorporating into mixed diets specific ingredients thought or known to have antidiarrheal properties, such as green banana [56,57]. A synthesis of the evidence for the full range of management options would best inform guidelines for optimal diet selection for childhood diarrhea treatment. It would also have been useful to investigate other dietary characteristics such as fibre content or osmolality, but there were insufficient data available across studies to enable this. Additionally, all but one [16] of the 29 studies that we included in our review were conducted in 1999 or earlier and only six studies [18,37,38,40,41,47] focused on persistent diarrhea. However, rather than a consequence of our review methods, we believe this limitation is almost certainly a consequence of the limited attention paid to childhood diarrhea in recent years.

## Conclusions

Continued feeding is important for limiting the nutritional consequences of decreased intake, digestion and absorption of essential nutrients during diarrheal illness. Among children in low- and middle-income countries, where the dual burden of diarrhea and malnutrition is greatest and where access to proprietary formulas and specialized ingredients is limited, continued breastfeeding should be encouraged and the use of locally available age-appropriate foods should be promoted for the majority of acute diarrhea cases. Lactose intolerance is an important complication in some cases, but even among those children for whom lactose avoidance may be necessary, nutritionally complete diets comprised of locally available ingredients can be used as least as effectively as commercial preparations or specialized ingredients. These same conclusions may also apply to the dietary management of children with persistent diarrhea, but the evidence remains limited. Overall, our review is supportive of current WHO/UNICEF recommendations for continued breastfeeding and administration of home-available age-appropriate foods to children 6 to 59 months of age with diarrhea.

## Competing interests

The authors declare that they have no competing interests.

## Authors’ contributions

ZAB conceived of the study; MFG, KW and ZAB wrote the protocol; MFG and KW conducted the literature searches, abstracted the data and assessed study quality, with DGB resolving any discrepancies; MFG analyzed the data and wrote the first draft of the manuscript; all authors revised the draft and approved the final manuscript.

## Supplementary Material

Additional File 1 Electronic search strategy for Medline, Embase and AMED databases Click here for file

Additional File 2 Abstracted data from all 29 studies included in the quantitative data synthesis Click here for file

Additional File 3 Eligible studies not included in the quantitative data synthesis Click here for file

Additional File 4 Forest plots for all comparisons and outcomes Click here for file

## Declarations

The publication costs for this supplement were funded by a grant from the Bill & Melinda Gates Foundation to the US Fund for UNICEF (grant 43386 to "Promote evidence-based decision making in designing maternal, neonatal, and child health interventions in low- and middle-income countries”). The Supplement Editor is the principal investigator and lead in the development of the Lives Saved Tool (LiST), supported by grant 43386. He declares that he has no competing interests.

This article has been published as part of *BMC Public Health* Volume 13 Supplement 3, 2013: The Lives Saved Tool in 2013: new capabilities and applications. The full contents of the supplement are available online at http://www.biomedcentral.com/bmcpublichealth/supplements/13/S3.
