# Supplementary material for: Dietary management of childhood diarrhea in low- and middle-income countries: a systematic review
Source: BMC Public Health. 2013 Sep 17;13(Suppl 3):S17. doi: 10.1186/1471-2458-13-S3-S17 (PMC3847348; doi:10.1186/1471-2458-13-S3-S17)
Supplement: Additional File 1 — Electronic search strategy for Medline, Embase and AMED databases [file 1471-2458-13-S3-S17-S1.docx]

| **Additional File 1 - Electronic search strategy for Medline, Embase and AMED databases** | |
| --- | --- |
|  |  |
|  |  |
| 1 | (diarrh$ or gastroenteritis).mp. |
| 2 | fe?d$.mp. |
| 3 | re?fe?d$.mp. |
| 4 | breast?fe?d$.mp. |
| 5 | food$.mp. |
| 6 | diet$.mp. |
| 7 | (newborn$ or neo?nat$ or infan$ or baby or babies or child$ or boy$ or girl$ or toddler$ or pre?school$ or p?ediatric$).mp. |
| 8 | exp Infant/ |
| 9 | exp Child/ |
| 10 | 1 and (2 or 3 or 4 or 5 or 6) and (7 or 8 or 9) |
| 11 | limit 10 to humans |
| 12 | random$.mp. |
| 13 | trial$.mp. |
| 14 | group$.mp. |
| 15 | compar$.mp. |
| 16 | limit 11 to (controlled clinical trial or randomized controlled trial) |
| 17 | 11 and (12 or 13 or 14 or 15) |
| 18 | 16 or 17 |
| 19 | limit 18 to "review" |
| 20 | 18 not 19 |
| 21 | 19 not 20 |
|  |  |
|  |  |
