# Supplementary material for: Dietary management of childhood diarrhea in low- and middle-income countries: a systematic review
Source: BMC Public Health. 2013 Sep 17;13(Suppl 3):S17. doi: 10.1186/1471-2458-13-S3-S17 (PMC3847348; doi:10.1186/1471-2458-13-S3-S17)
Supplement: Additional File 4 — Forest plots for all comparisons and outcomes [file 1471-2458-13-S3-S17-S4.pptx]

## Slide 1
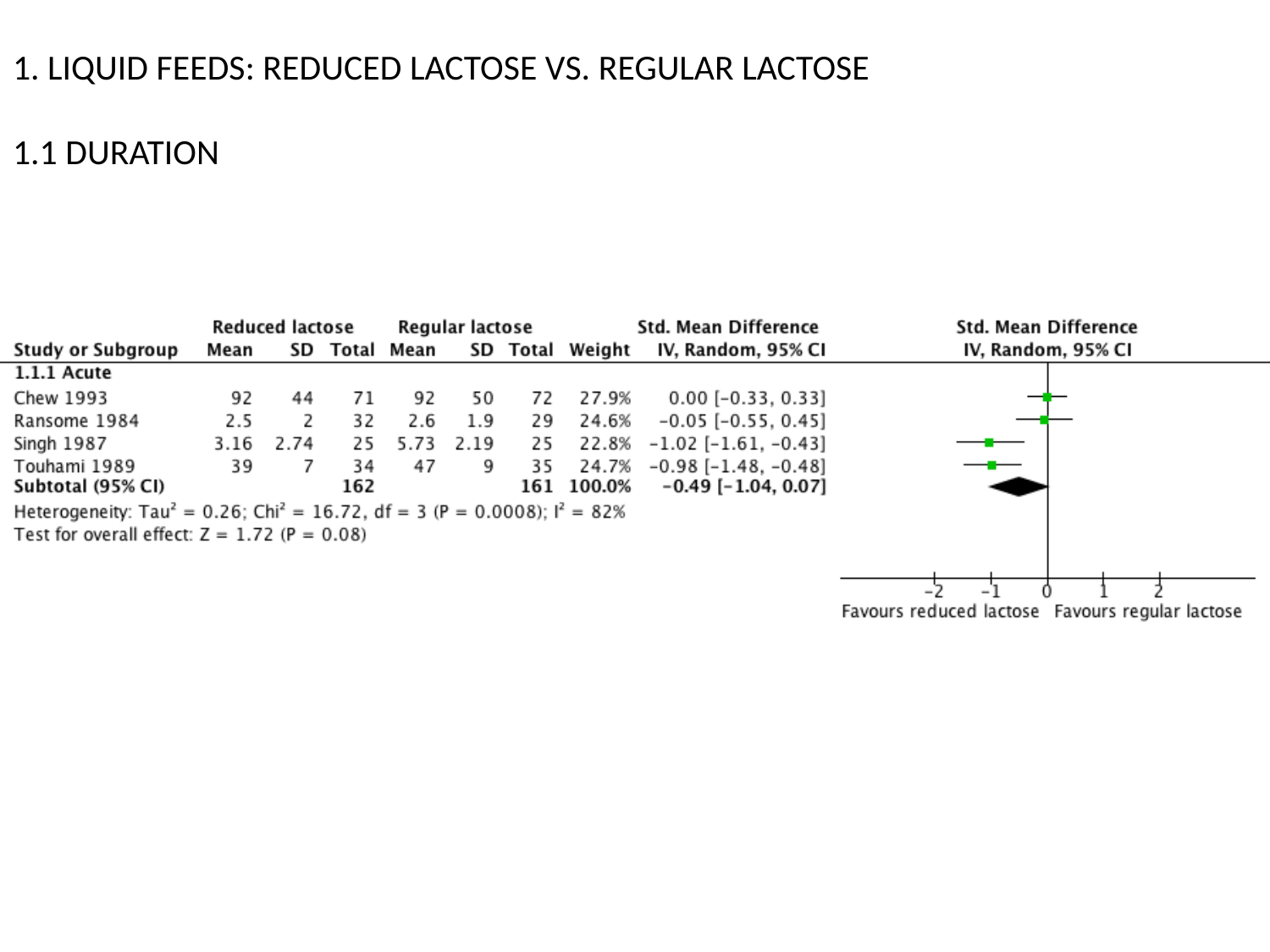

# 1. LIQUID FEEDS: REDUCED LACTOSE VS. REGULAR LACTOSE 1.1 DURATION

## Slide 2
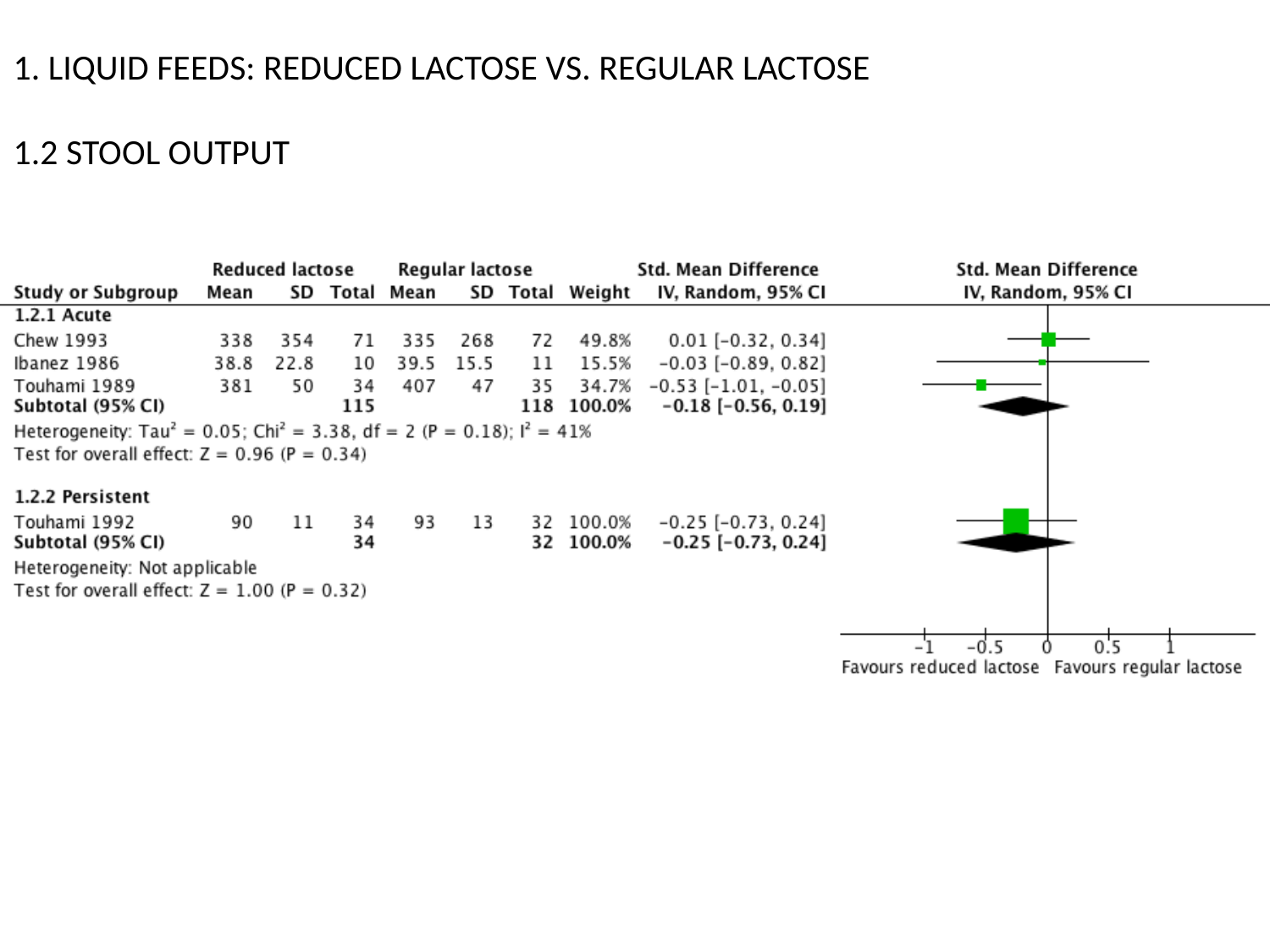

# 1. LIQUID FEEDS: REDUCED LACTOSE VS. REGULAR LACTOSE 1.2 STOOL OUTPUT

## Slide 3
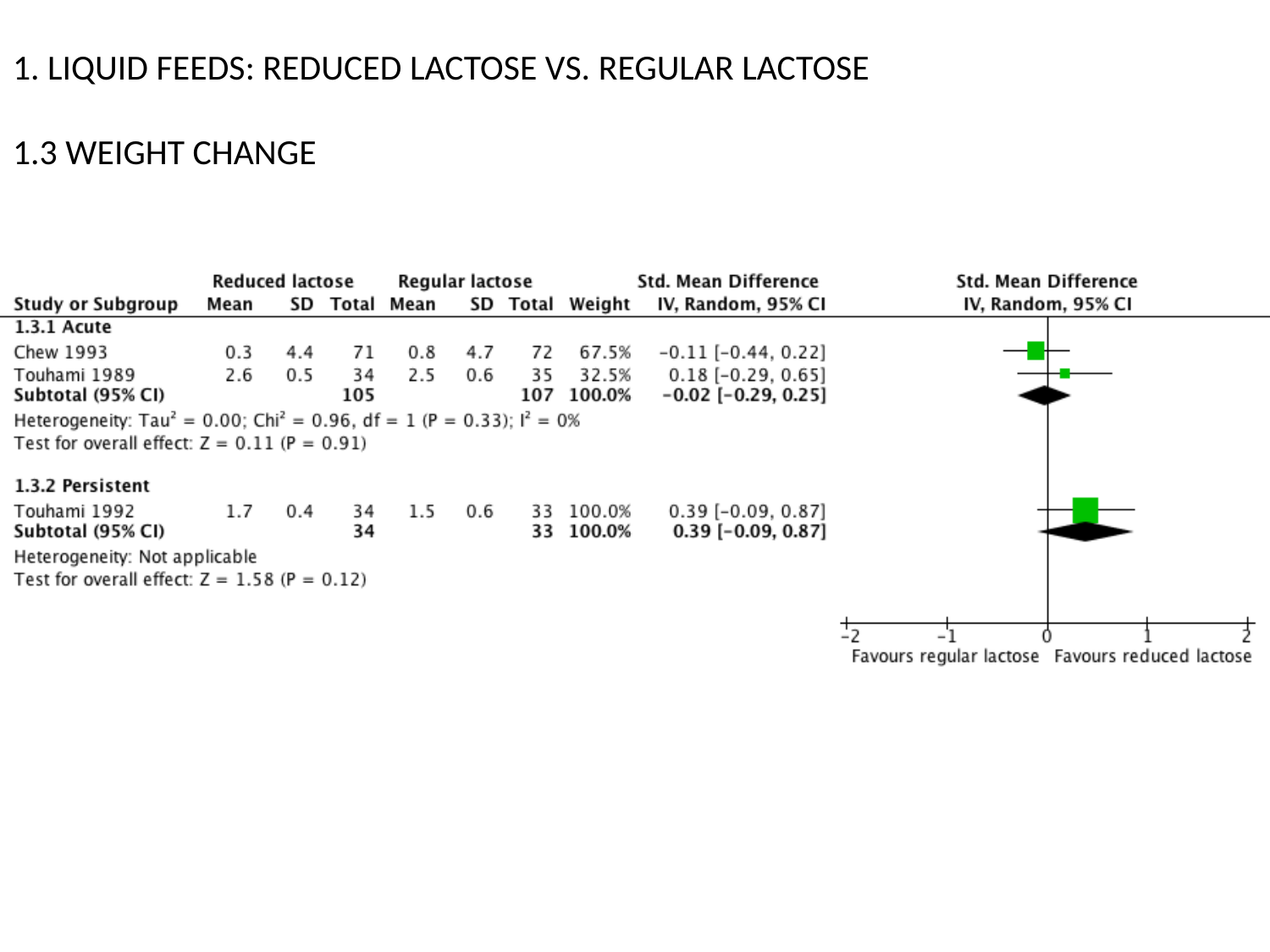

# 1. LIQUID FEEDS: REDUCED LACTOSE VS. REGULAR LACTOSE 1.3 WEIGHT CHANGE

## Slide 4
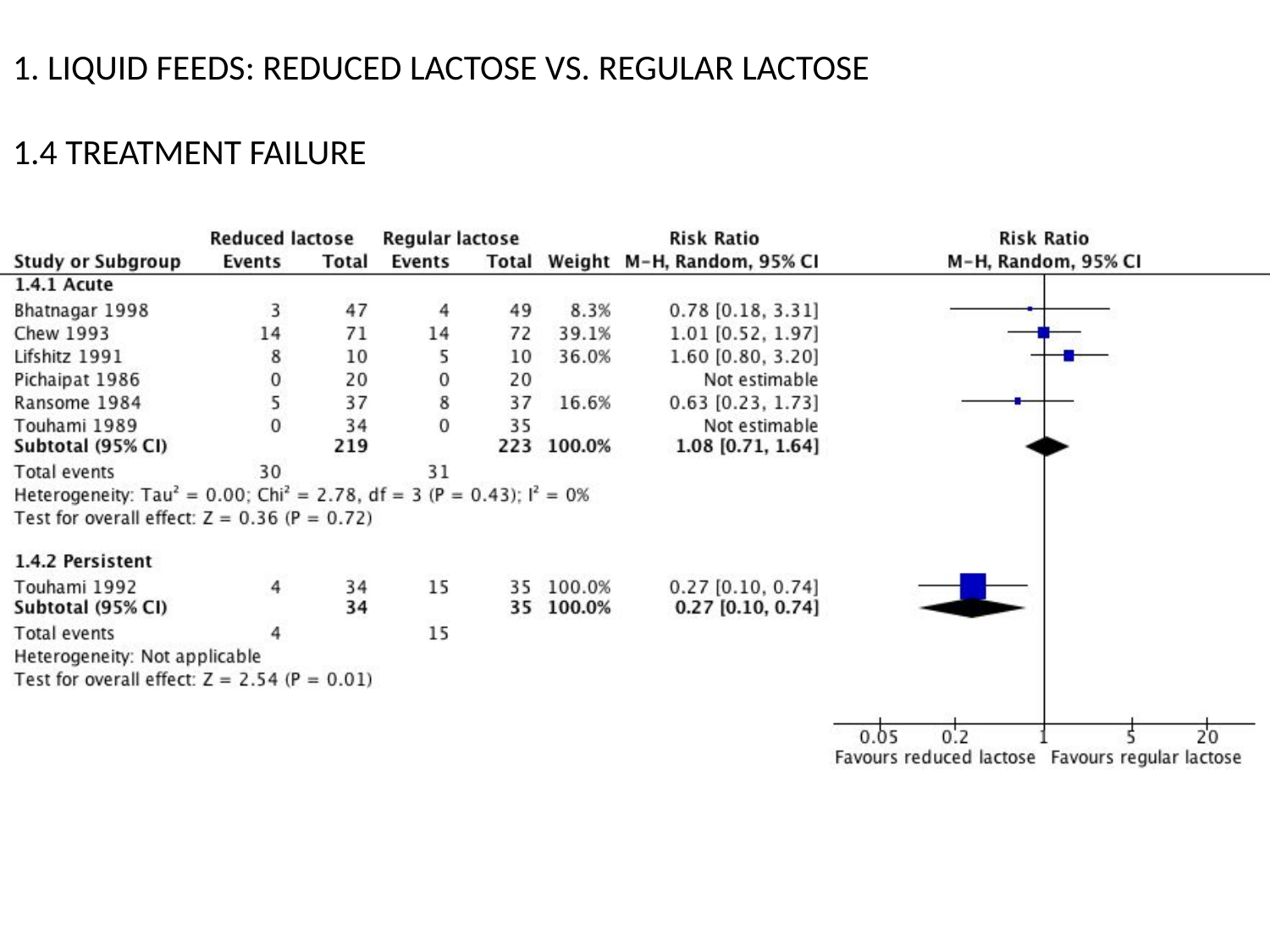

# 1. LIQUID FEEDS: REDUCED LACTOSE VS. REGULAR LACTOSE 1.4 TREATMENT FAILURE

## Slide 5
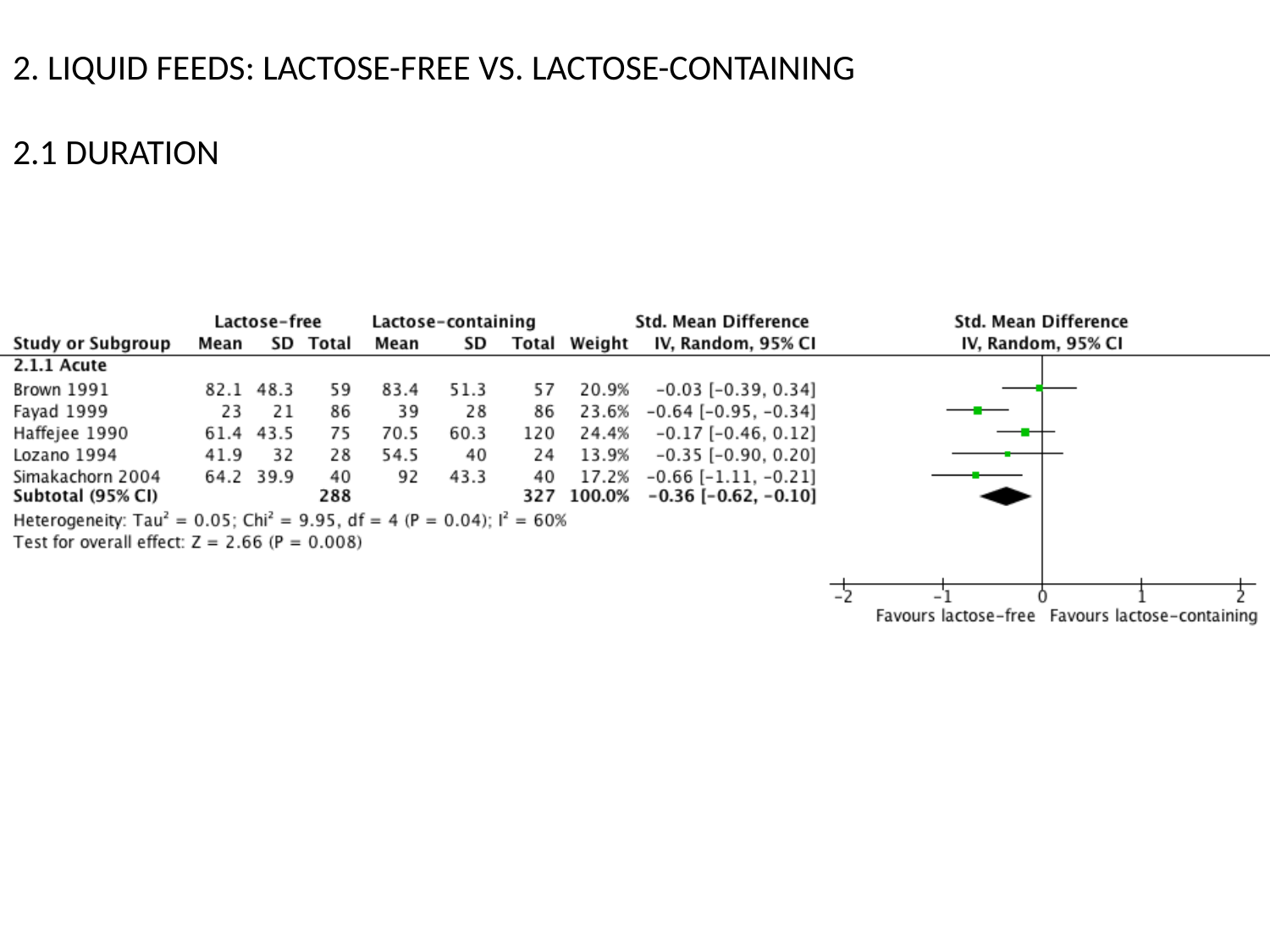

# 2. LIQUID FEEDS: LACTOSE-FREE VS. LACTOSE-CONTAINING 2.1 DURATION

## Slide 6
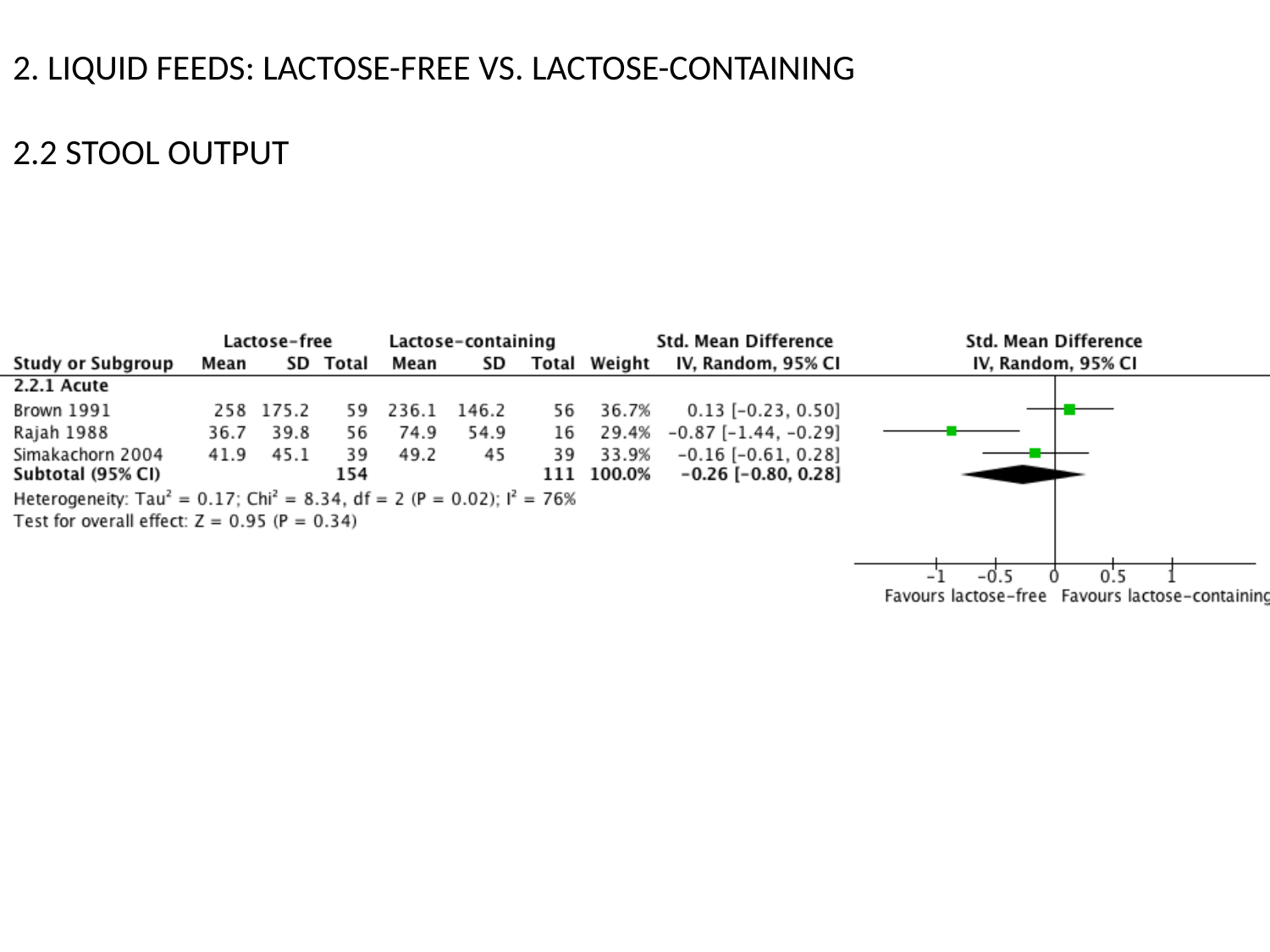

# 2. LIQUID FEEDS: LACTOSE-FREE VS. LACTOSE-CONTAINING 2.2 STOOL OUTPUT

## Slide 7
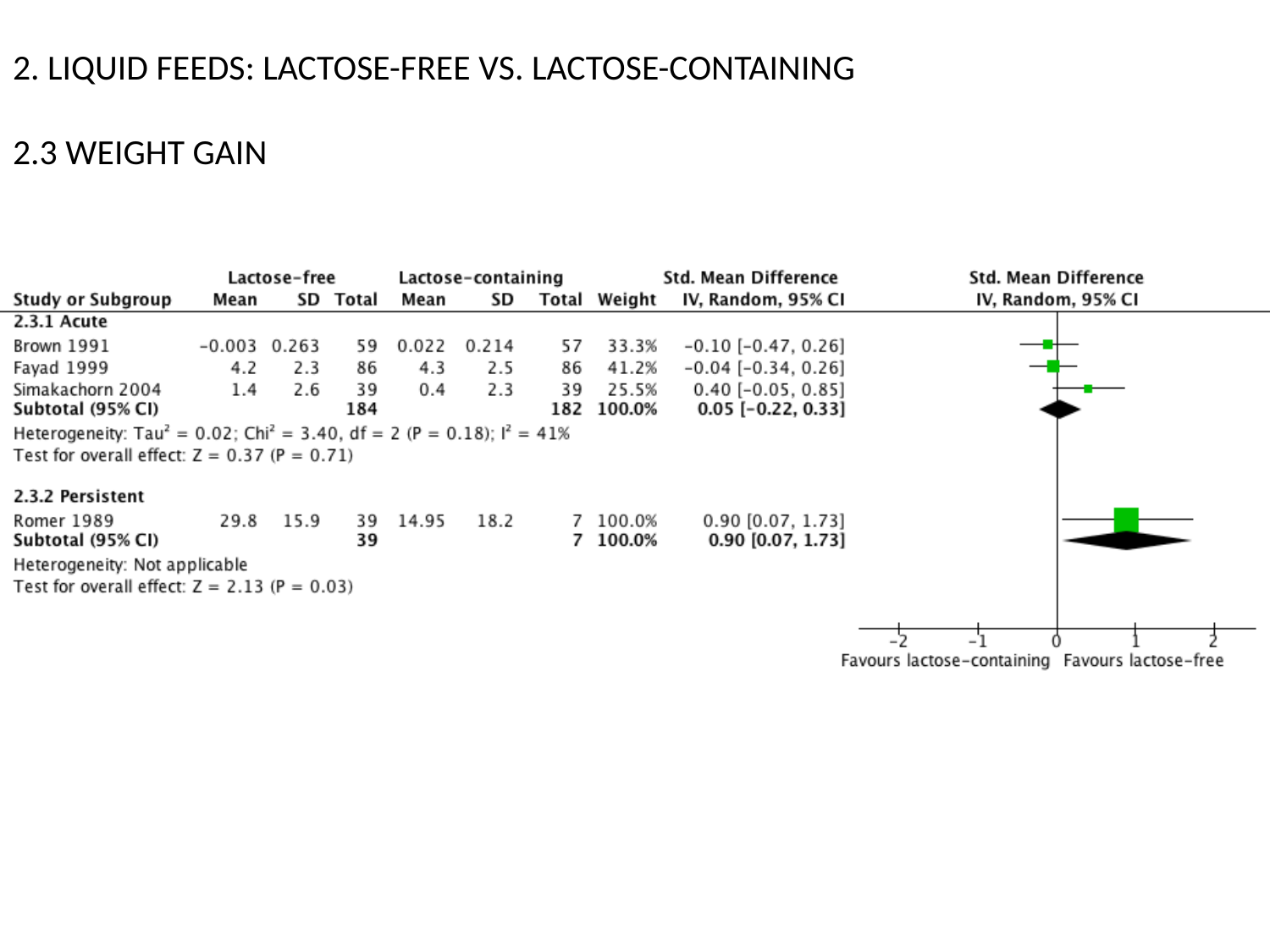

# 2. LIQUID FEEDS: LACTOSE-FREE VS. LACTOSE-CONTAINING 2.3 WEIGHT GAIN

## Slide 8
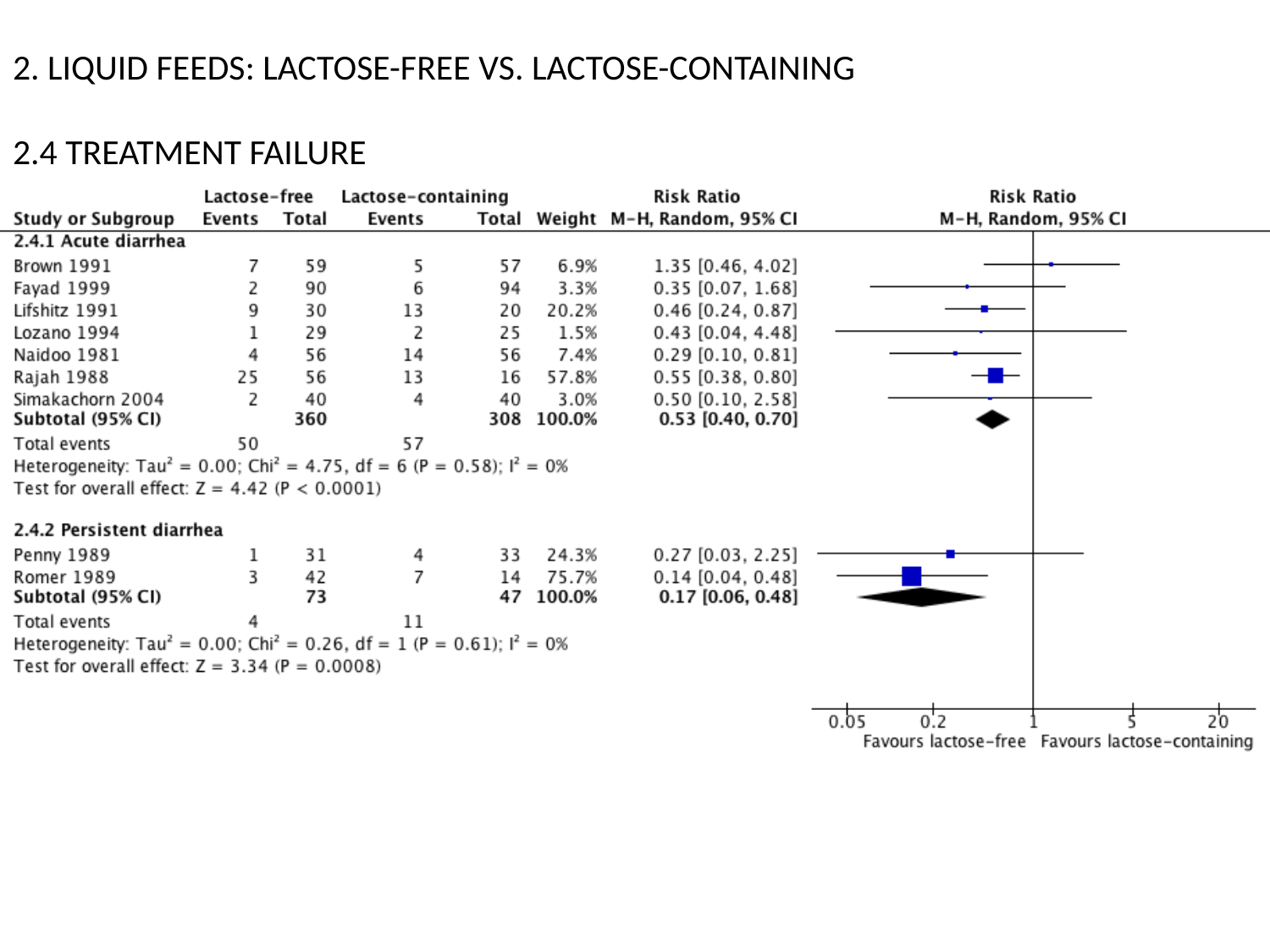

# 2. LIQUID FEEDS: LACTOSE-FREE VS. LACTOSE-CONTAINING 2.4 TREATMENT FAILURE

## Slide 9
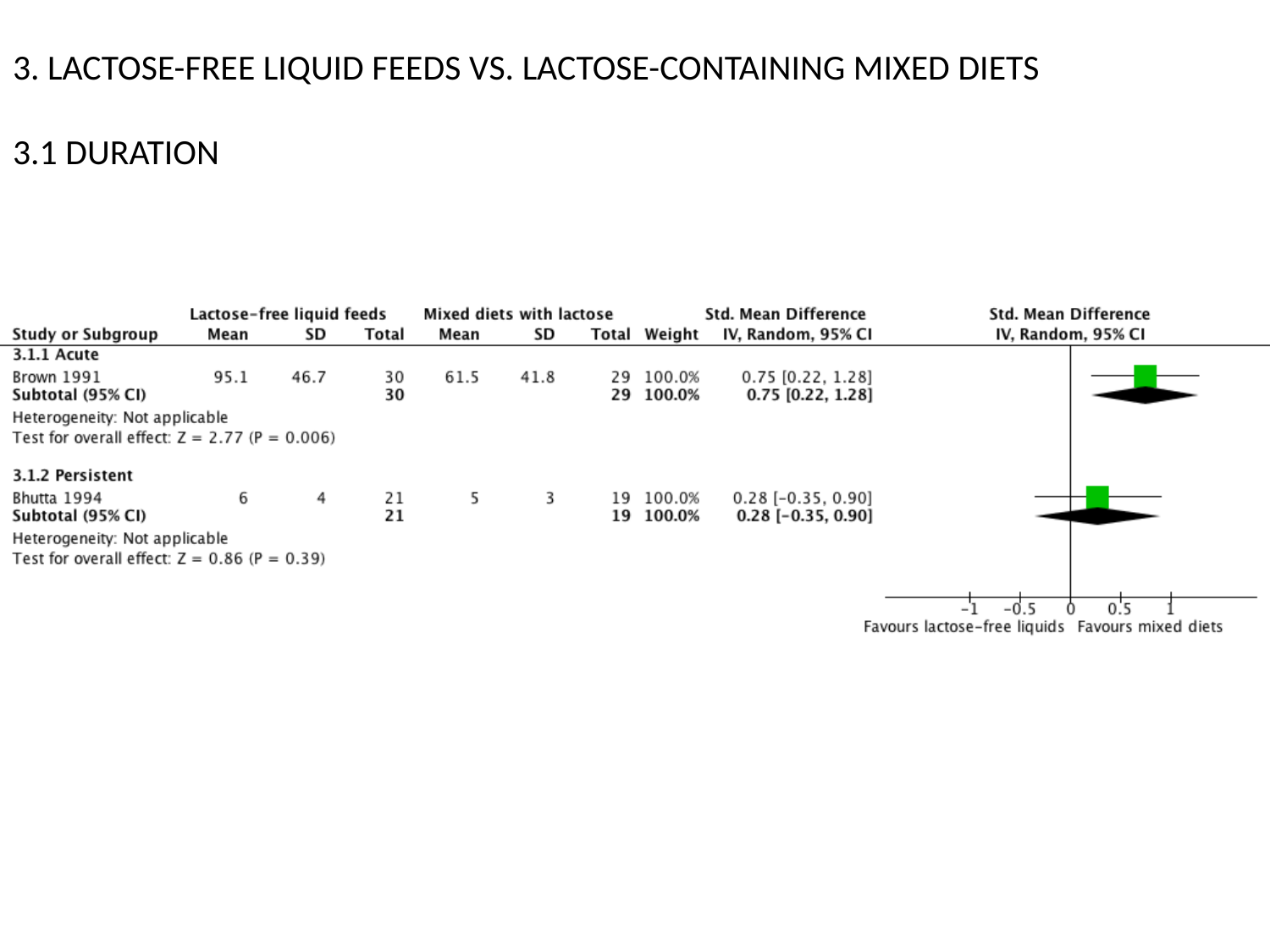

# 3. LACTOSE-FREE LIQUID FEEDS VS. LACTOSE-CONTAINING MIXED DIETS 3.1 DURATION

## Slide 10
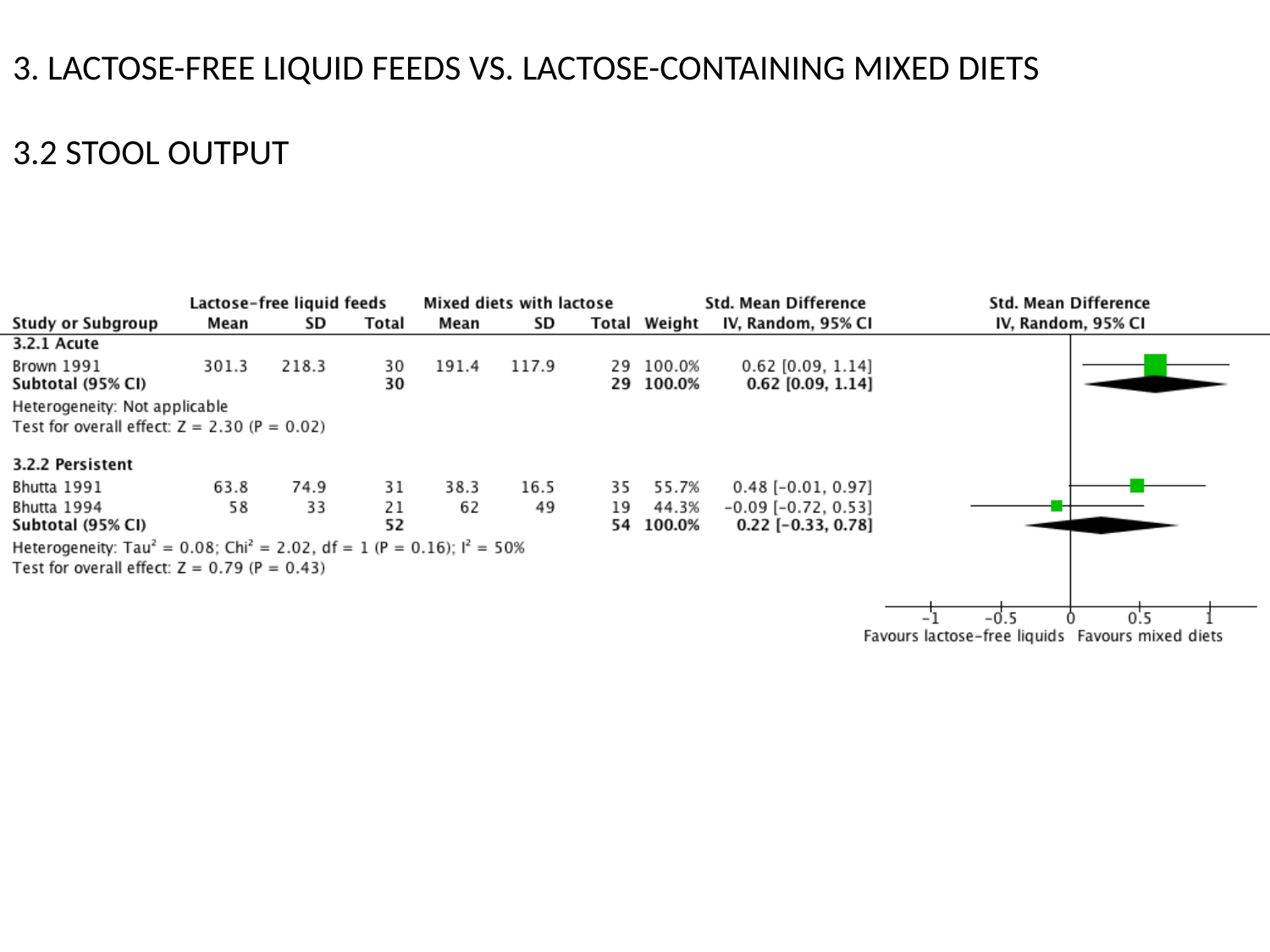

# 3. LACTOSE-FREE LIQUID FEEDS VS. LACTOSE-CONTAINING MIXED DIETS 3.2 STOOL OUTPUT

## Slide 11
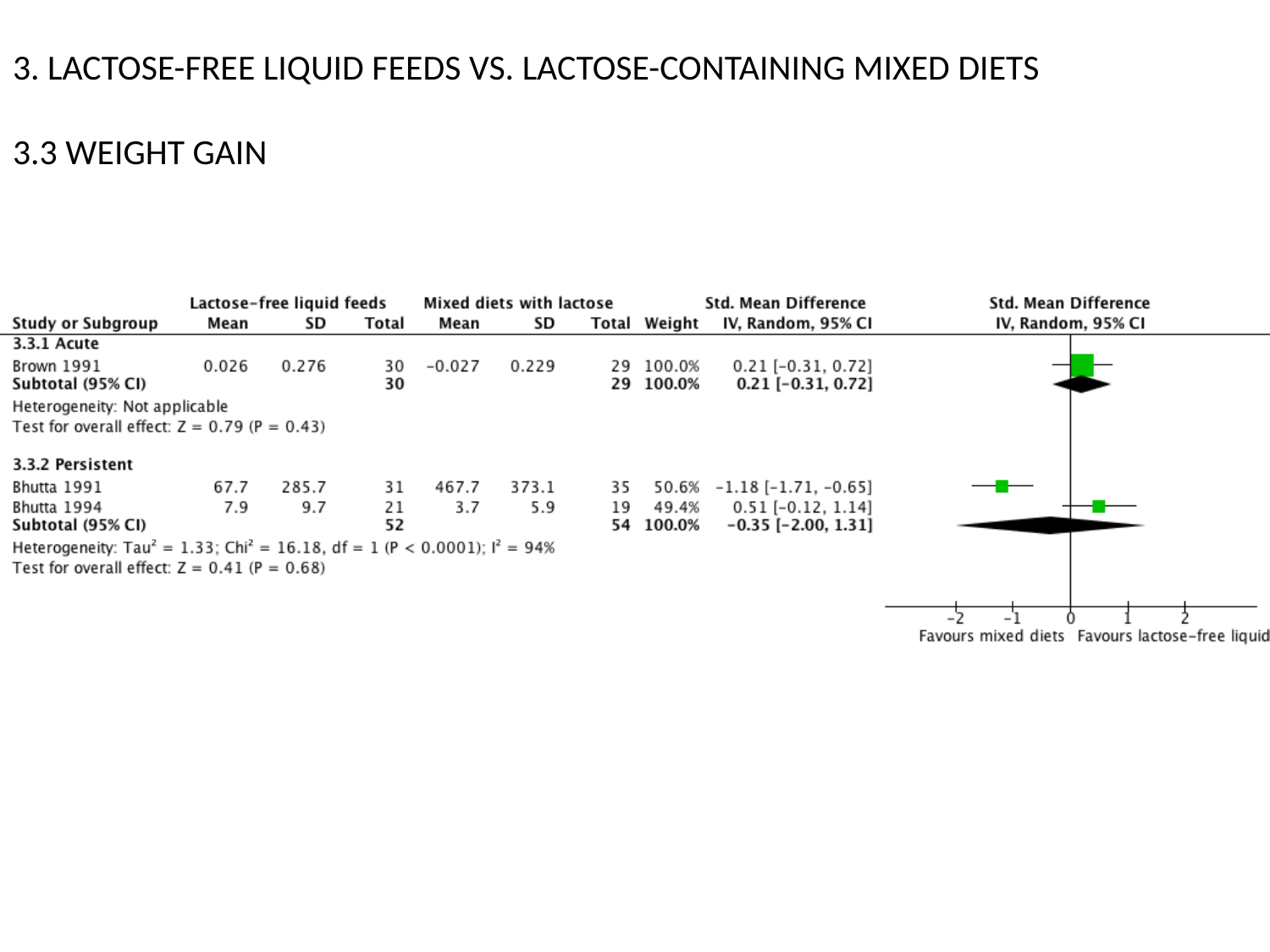

# 3. LACTOSE-FREE LIQUID FEEDS VS. LACTOSE-CONTAINING MIXED DIETS 3.3 WEIGHT GAIN

## Slide 12
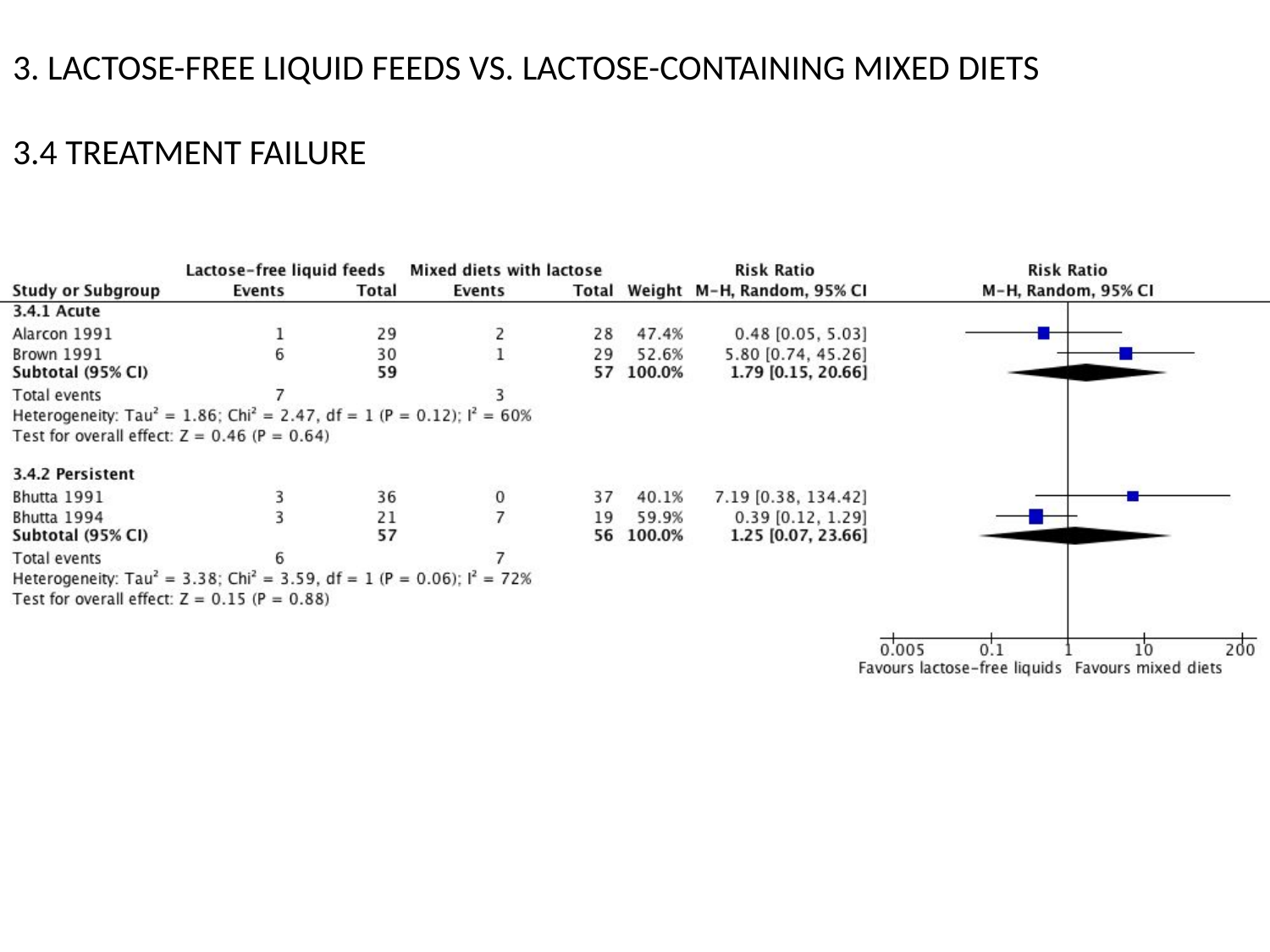

# 3. LACTOSE-FREE LIQUID FEEDS VS. LACTOSE-CONTAINING MIXED DIETS 3.4 TREATMENT FAILURE

## Slide 13
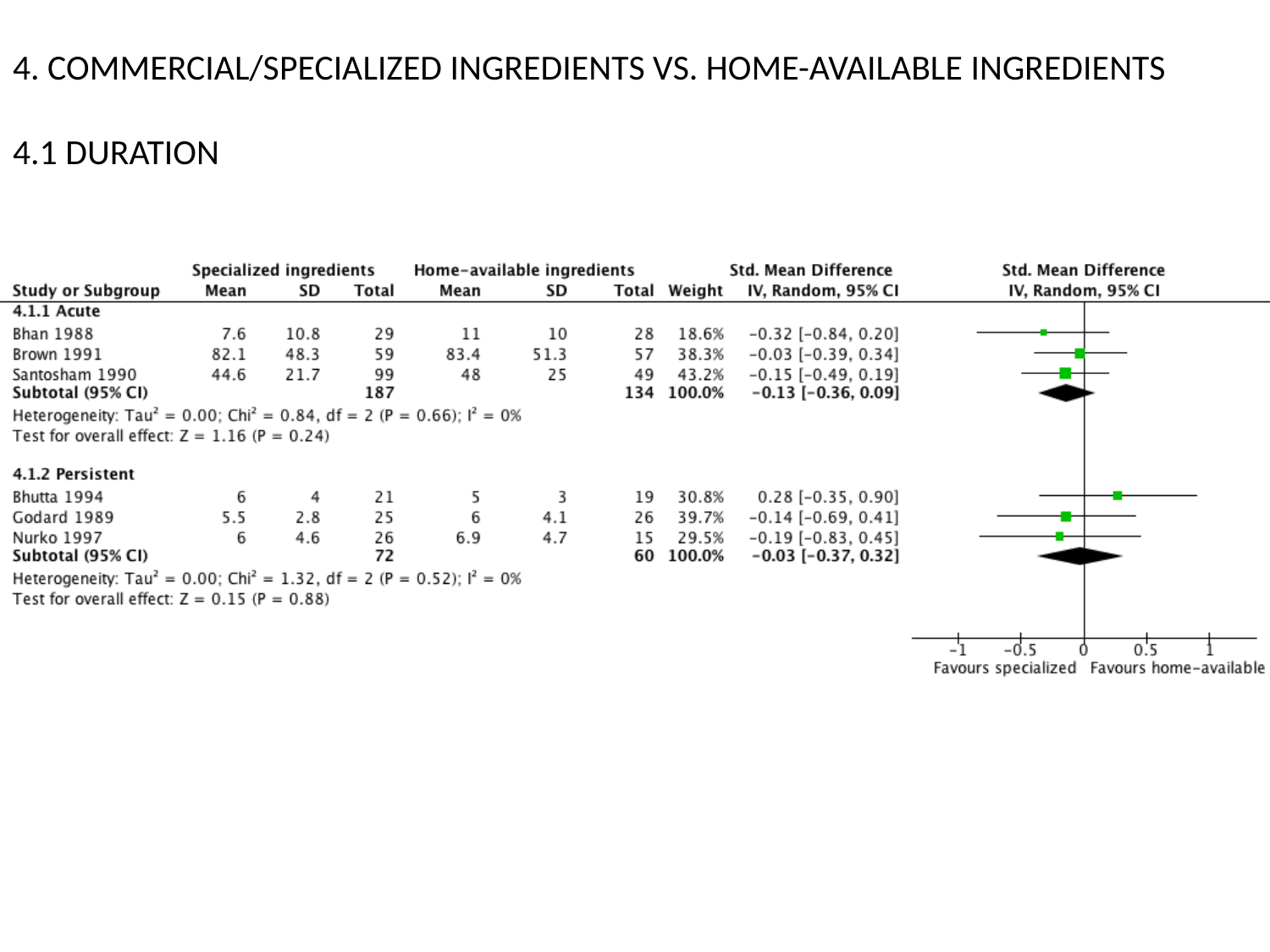

# 4. COMMERCIAL/SPECIALIZED INGREDIENTS VS. HOME-AVAILABLE INGREDIENTS 4.1 DURATION

## Slide 14
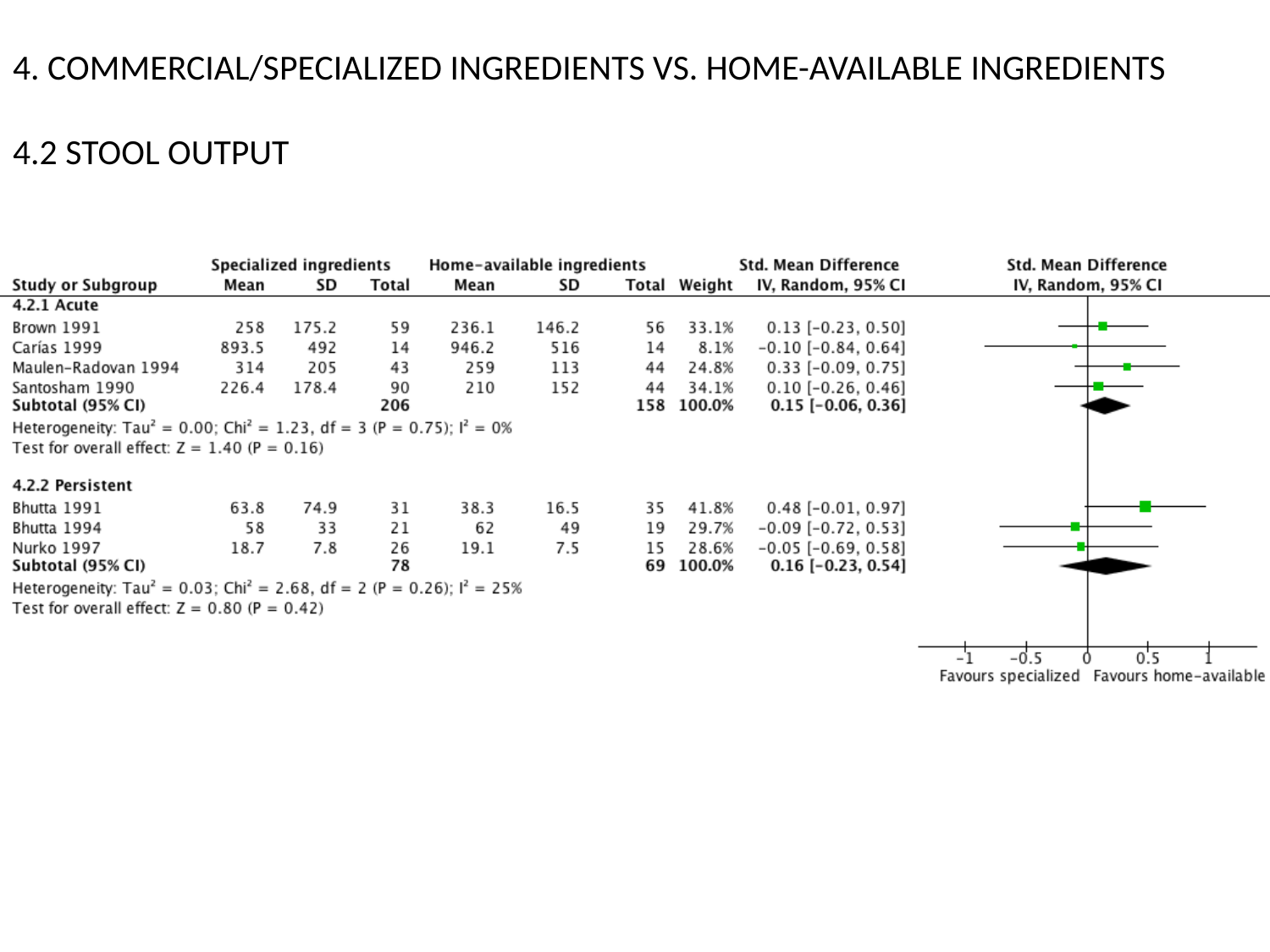

# 4. COMMERCIAL/SPECIALIZED INGREDIENTS VS. HOME-AVAILABLE INGREDIENTS 4.2 STOOL OUTPUT

## Slide 15
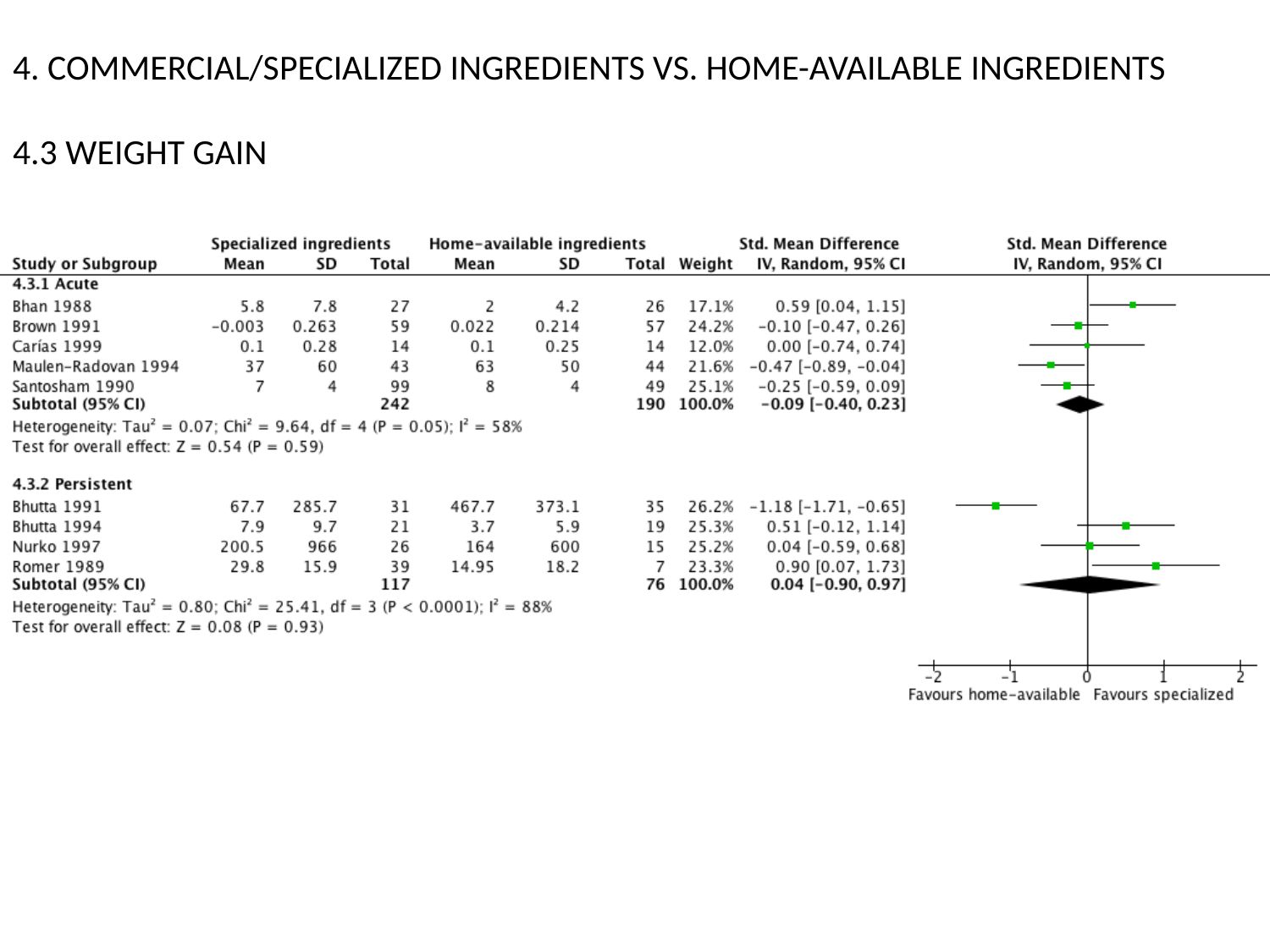

# 4. COMMERCIAL/SPECIALIZED INGREDIENTS VS. HOME-AVAILABLE INGREDIENTS 4.3 WEIGHT GAIN

## Slide 16
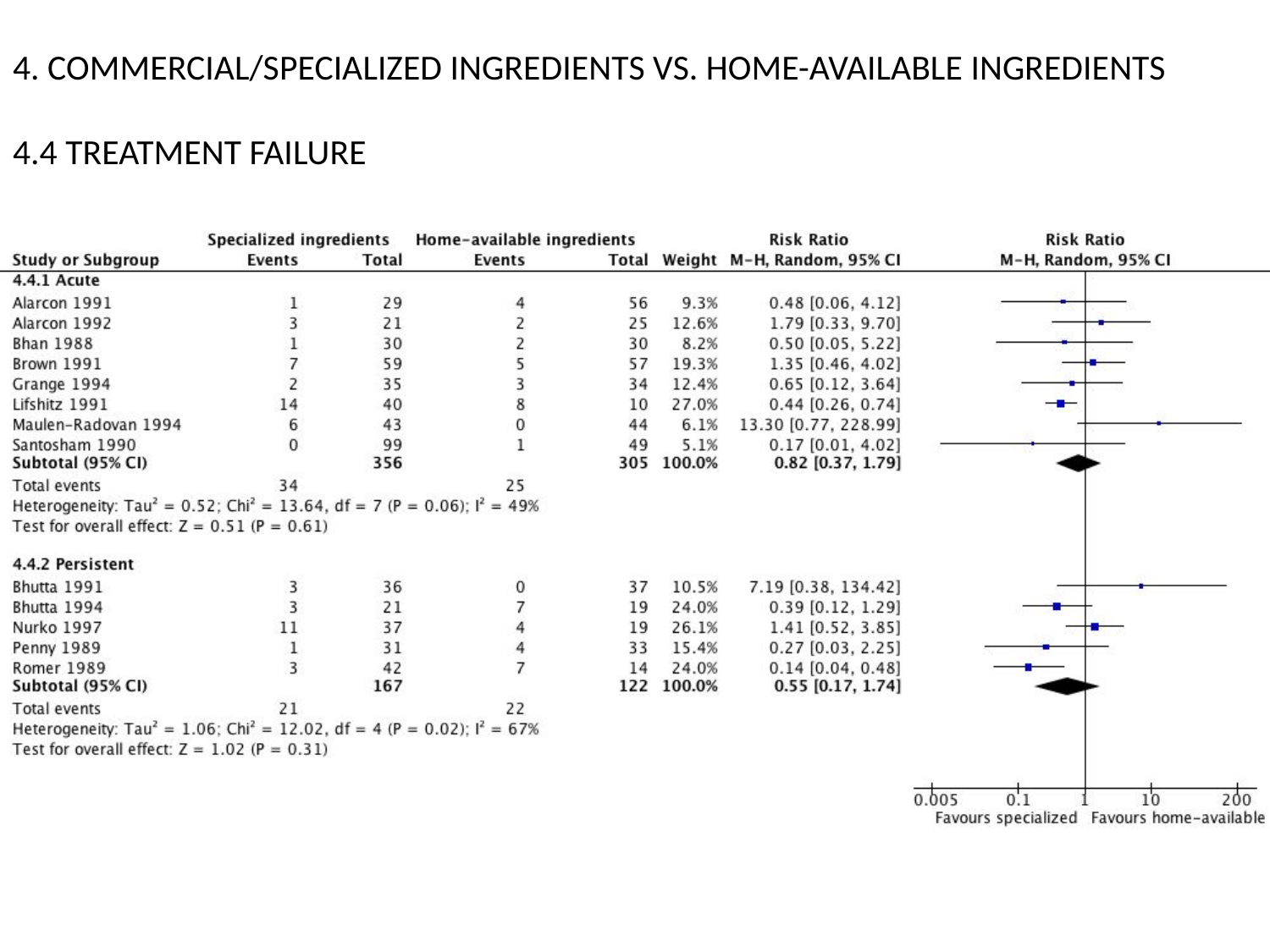

# 4. COMMERCIAL/SPECIALIZED INGREDIENTS VS. HOME-AVAILABLE INGREDIENTS 4.4 TREATMENT FAILURE

## Slide 17
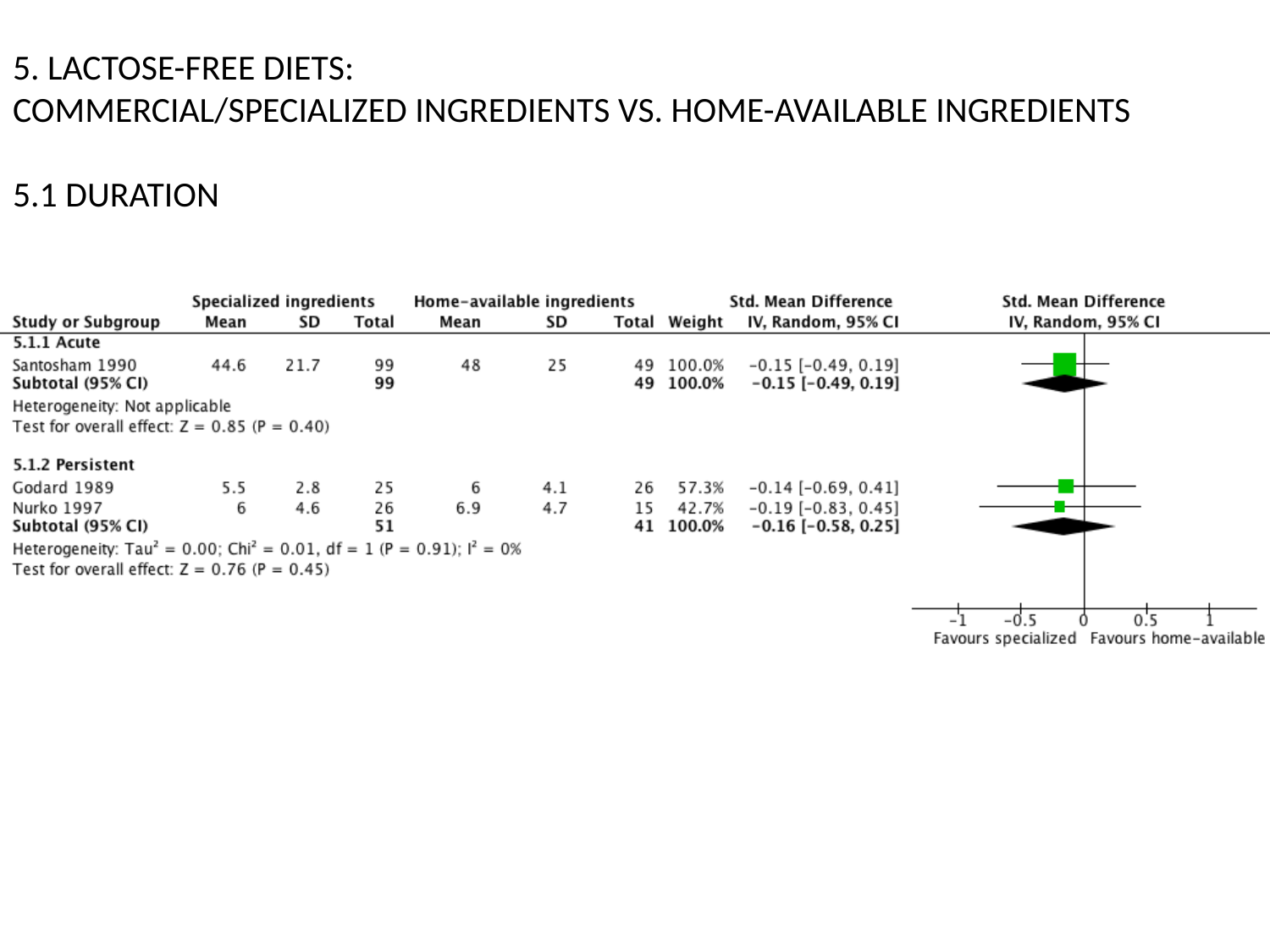

# 5. LACTOSE-FREE DIETS: COMMERCIAL/SPECIALIZED INGREDIENTS VS. HOME-AVAILABLE INGREDIENTS 5.1 DURATION

## Slide 18
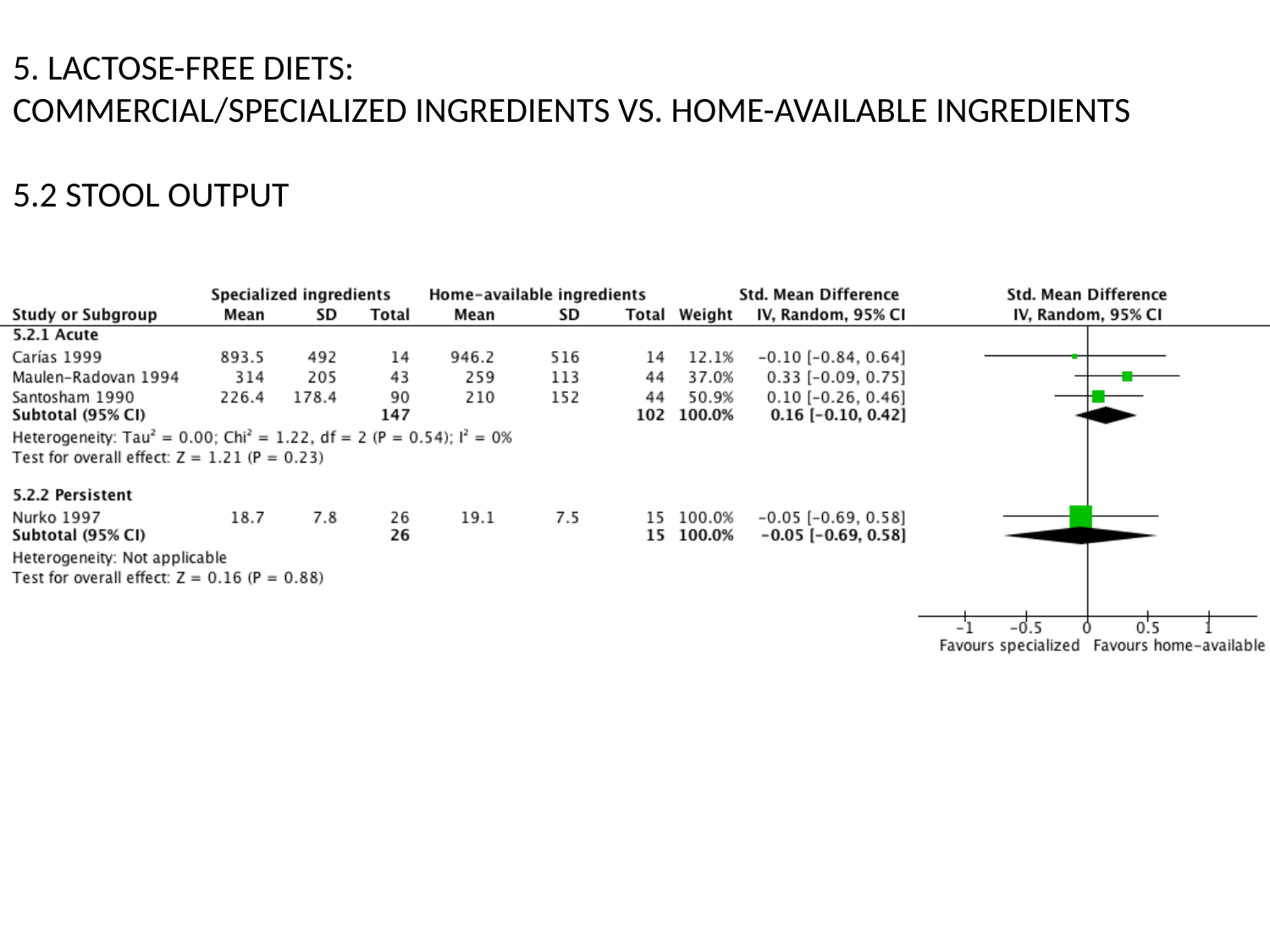

# 5. LACTOSE-FREE DIETS: COMMERCIAL/SPECIALIZED INGREDIENTS VS. HOME-AVAILABLE INGREDIENTS 5.2 STOOL OUTPUT

## Slide 19
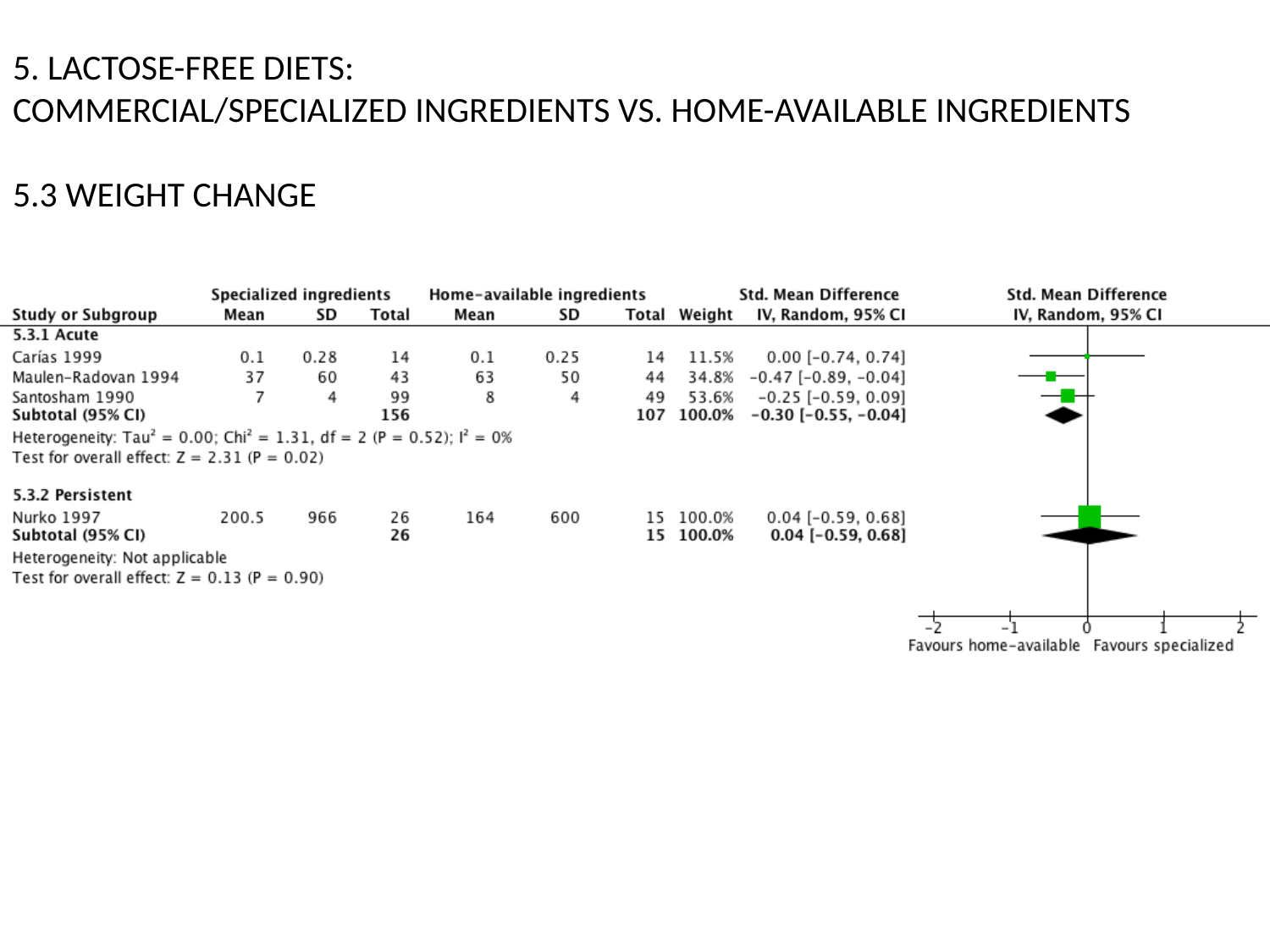

# 5. LACTOSE-FREE DIETS: COMMERCIAL/SPECIALIZED INGREDIENTS VS. HOME-AVAILABLE INGREDIENTS 5.3 WEIGHT CHANGE

## Slide 20
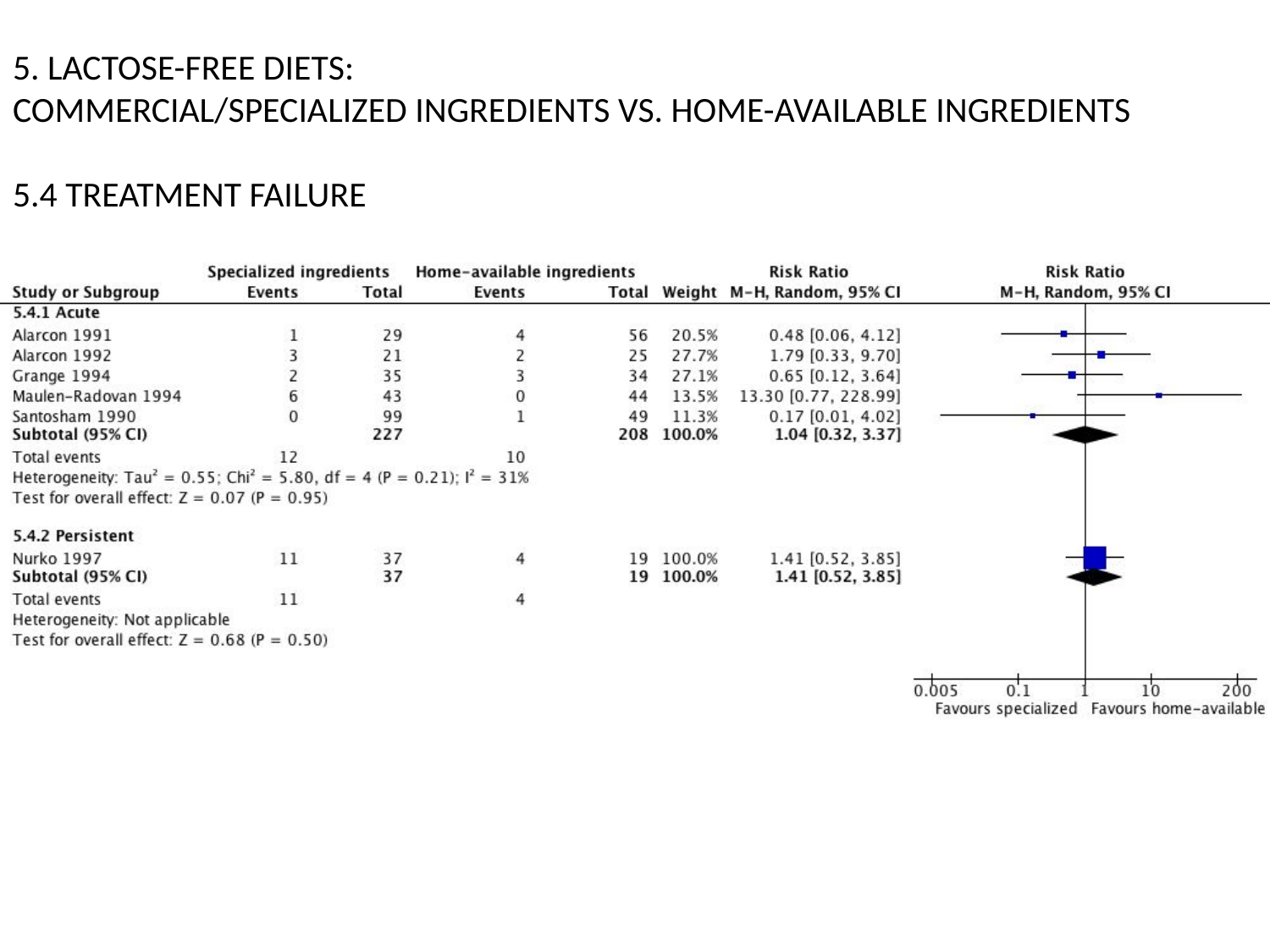

# 5. LACTOSE-FREE DIETS: COMMERCIAL/SPECIALIZED INGREDIENTS VS. HOME-AVAILABLE INGREDIENTS 5.4 TREATMENT FAILURE
